# Pharmacodynamic Evaluation of Adjuvant Targets: Low Molecular Weight PBP7/8 Effects on β-Lactam Activity Against Carbapenem-Resistant Acinetobacter Baumannii

**DOI:** 10.3390/ph18060918

**Published:** 2025-06-18

**Authors:** Brian M. Ho, Jingxiu Jin, Jacob T. Sanborn, Thomas D. Nguyen, Navaldeep Singh, Christina Cheng, Nader N. Nasief, Ulrike Carlino-MacDonald, Brian T. Tsuji, Yanan Zhao, Liang Chen, Bartolome Moya, Thomas A. Russo, Nicholas M. Smith

**Affiliations:** 1 Department of Pharmacy Practice, School of Pharmacy and Pharmaceutical Sciences, University at Buffalo, The State University of New York, Buffalo, NY 14214, USA; bmho@buffalo.edu (B.M.H.); jingxiuj@buffalo.edu (J.J.); jtsanbor@buffalo.edu (J.T.S.); tn34@buffalo.edu (T.D.N.); navaldee@buffalo.edu (N.S.); ccheng35@buffalo.edu (C.C.); nnn2@buffalo.edu (N.N.N.); btsuji@buffalo.edu (B.T.T.); yananzh@buffalo.edu (Y.Z.); liangch@buffalo.edu (L.C.); 2 Department of Medicine, Jacobs School of Medicine and Biomedical Sciences, University at Buffalo, The State University of New York, Buffalo, NY 14203, USA; ucarlino@buffalo.edu (U.C.-M.); trusso@buffalo.edu (T.A.R.); 3 Veterans Administration Western New York Healthcare System, Buffalo, NY 14215, USA; 4 Servicio de Microbiología and Unidad de Investigación, Hospital Universitario Son Espases, Instituto de Investigación Sanitaria Illes Balears (IdISBa), 07120 Palma, Balearic Islands, Spain; tomeu.moya@idisba.es; 5 Centro de Investigación Biomédica en Red en Enfermedades Infecciosas (CIBERINFEC), 07120 Palma, Balearic Islands, Spain; 6 The Witebsky Center for Microbial Pathogenesis, University at Buffalo, The State University of New York, Buffalo, NY 14203, USA

**Keywords:** acinetobacter baumannii, antibiotic resistance, low molecular weight penicillin binding proteins

## Abstract

**Background/Objectives:** The increasing occurrence of carbapenem resistance *A. baumannii* (CRAB) has forced clinicians to seek out alternative options with activity against CRAB. CRAB with inactivated PBP7/8 has been shown to result in an increased outer membrane permeability and could serve as a potential new adjuvant target. **Methods**: Two isogenic clinical isolates of *A. baumannii* HUMC1 were utilized (WT and HUMC1 ΔPBP7/8). Static concentration time-kill assays were performed against both isolates with escalating exposures to antibiotics. The resulting data were modeled using the Monolix software suite to capture parameters related to bacterial killing and PBP7/8 synergism. The model results were used to prospectively simulate clinically relevant antibiotic dosing of three antibiotics under physiological conditions and were validated using a hollow-fiber infection model (HFIM). **Results**: Treatment with monotherapy or combination therapy resulted in concentration-dependent killing for both isolates. Bacterial killing was greater with HUMC1 ΔPBP7/8 for all tested antibiotic concentrations. The mean bacterial population reduction was 4.38 log_10_ CFU/mL for HUMC1 and 5.38 log_10_ CFU/mL for HUMC1ΔPBP7/8 knockout isolate. The final mechanism-based model demonstrated improved antibacterial activity with PBP7/8 inhibition through a decline in KC_50_ values of 59.7% across the beta-lactams in the PBP7/8 knockout. HFIM observations that were retrospectively compared to the simulated model-predicted bacterial concentration time course showed our final model was able to appropriately capture changes in bacterial population within a dynamic HFIM scenario. **Conclusions**: The quantification of KC_50_ decline and increase in effectiveness of previously sidelined antimicrobial therapies with PBP7/8 inhibition suggests PBP7/8 is a promising potential target for an antibacterial adjuvant. This lends further support to advance to next-stage studies for identifying compounds that specifically inhibit PBP7/8 activity.

## 1. Introduction

The continuing rise of antimicrobial resistance in *Acinetobacter baumannii* is quickly becoming an urgent health threat [1]. The number of CRAB infections continues to increase, especially notable during the COVID-19 pandemic, with high incidence among immunocompromised patients as well as individuals undergoing extended hospital stays [2,3]. *A. baumannii* has a natural competency to acquire resistance genes to many of the commonly used antimicrobial classes [4]. As a result, the Centers for Disease Control and Prevention have classified carbapenem-resistant *A. baumannii* (CRAB) as a top priority among pathogens of interest [5]. CRAB is of particular concern as it often combines multiple resistance mechanisms which can function synergistically to impair the efficacy of the few remaining available antibiotic options [6]. The increasing occurrence of carbapenem resistance has forced clinicians to seek out alternative antibiotic classes with activity against *A. baumannii*, such as the polymyxins [7]. However, polymyxins have a higher risk of nephrotoxicity and generally lower clinical efficacy compared with other antibiotics. Unfortunately, there is now additional evidence that resistance is emerging towards this last-resort antimicrobial class [8,9]. With a dearth of new antibiotics in development, new and alternative strategies for combatting these extensively drug-resistant *A. baumannii* strains should be more thoroughly considered [10,11].

One proposed strategy for combatting drug resistance in *A. baumannii* is through the exploitation of low molecular weight penicillin binding proteins (LMW PBPs). Such enzymes play an important role in the mediation of peptidoglycan synthesis, which in turn is critical for bacterial survival [12]. Bacterial viability requires functionality of one high molecular weight (HMW) Class A and one HMW Class B PBP. By comparison, the loss of *all* LMW PBPs may not be lethal [13]. Therefore, high molecular weight PBPs have traditionally been the focus of antimicrobial development as they had a greater chance of achieving treatment success, making them the target of our current beta-lactam suite [14].

Previous studies exploring LMW PBP7/8 in *A. baumannii* have confirmed that PBP7/8 was not an essential gene for in vitro survival under rich medium but was essential ex vivo in human ascites and serum [15]. Further exploration demonstrated that PBP7/8 loss produced a compromised outer membrane in *A. baumannii*, which may be leveraged to enhance target site penetration of concomitantly administered beta-lactam therapy [16]. Additionally, inactivation of PBP7/8 may increase membrane permeability to work synergistically with other antimicrobials to increase target site concentrations (e.g., beta-lactams) or reduce the necessary dose of LPS-binding permeabilizing agents (e.g., polymyxins) [16]. However, there is a lack of available supporting information quantifying the effect of PBP7/8 inhibition on the intrinsic activity of clinically relevant antimicrobials and on the presence of any additional synergistic effects from administering antibiotics of different classes.

This study utilizes a novel pharmacodynamic-led evaluation of adjuvant targets (PEAT) strategy to study the viability for PBP7/8 to serve as an adjuvant target. Adjuvants have nominal standalone activity, making the assessment of adjuvant targets challenging without having an existing active antibiotic pre-selected. Furthermore, assessing possible adjuvant targets for clinical viability is improved through the utilization of clinically relevant concentrations of partner antibiotics.

Our objective was to demonstrate and quantify the extent to which PBP7/8 inactivation, as a surrogate for a small molecule inhibitor with “ideal” activity, can enhance the activity of HMW-targeting beta-lactams against CRAB. As a comparator, polymyxin B (PMB) was utilized as it has a long history of use in combination with other agents for hypothesized LPS-binding membrane permeabilization effects [17]. Combination therapies consisting of a beta-lactam and polymyxin B were performed to further explore a potential “triple therapy” utilizing two distinct mechanisms of inducing membrane permeabilization. Pharmacodynamics were then characterized utilizing mechanism-based modeling of the in vitro static concentration time-kill data. The secondary objective was to demonstrate that the PEAT strategy was predictive under dynamic dosing conditions longitudinally, which was prospectively validated using a 7-day hollow-fiber infection model (HFIM) study.

## 2. Results

### 2.1. Static Concentration Time-Kill Experiments

Data from 16 monotherapy arms and 48 combination arms consisting of a beta-lactam plus polymyxin B for each wild-type HUMC1 and HUMC1ΔPBP7/8 knockout isolate was included. Treatment with monotherapy or combination therapy resulted in killing that was concentration-dependent for both isolates. HUMC1ΔPBP7/8 experienced greater bacterial killing compared with the clinical wild-type strain under all tested mono- and combination therapy conditions. Mean bacterial population reduction, regardless of antibiotic, was 4.38 log_10_ CFU/mL for HUMC1 and 5.38 log_10_ CFU/mL for HUMC1ΔPBP7/8 knockout isolate. Under monotherapy conditions for the duration of the experiment, neither the WT nor HUMC1ΔPBP7/8 knockout isolates were able to recover to the growth control concentration. Regarding the combination therapy arms, all tested combination therapies against both WT and HUMC1ΔPBP7/8 knockout isolates remained below the 7 log_10_ CFU/mL starting inoculum concentration, with many combination therapy arms experiencing a population decline below the limit of quantification.

### 2.2. Mechanism-Based Model of PBP7/8 Effects

The final model implemented a two-subpopulation structure for antibiotic-susceptible and resistant cells. The covariate effects of PBP7/8 knockout status were utilized to describe a systematic change in the concentration of antibiotic required for half-maximal killing (KC_50_) for each individual drug. The parameter estimates from the model indicate that for all antibiotic therapies, the killing activity in the HUMC1ΔPBP7/8 knockout strain was greater than that of the wild-type clinical isolate strain. Improved activity was demonstrated by the decline in KC_50_ values for all five antibiotic therapies in the PBP7/8 knockout strain as shown in the final model parameter estimates in Table 1. The average decline of KC_50_ values across the tested beta-lactams due to PBP7/8 knockout was 59.7% while for polymyxin B it was 39.0%. %RSE values less than 20% provide support regarding the confidence of the parameter estimates.

**Figure 1:**
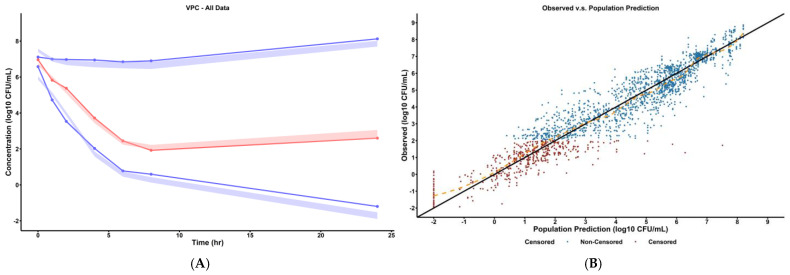
(**A**) **Visual predictive check of final pharmacodynamic model.** The solid points represent the mean observed bacterial concentrations at the marked time. The simulated prediction intervals for the 10th, 50th, and 90th percentile are displayed as the colored areas (red for 50th percentile and blue for 10th and 90th percentiles) as predicted by the final mechanism-based model. Refer to supplement Figure S3 for VPC plots stratified by dosing scheme. (**B**) **Observed versus predicted plot of final pharmacodynamic model.** The solid line represents the line of identity and the dashed orange line represents the spline line indicating the general trend of the data. Refer to Figures S1 and S2 for goodness-of-fit plots (observed vs. predicted, PWRES vs. population prediction) stratified by dosing scheme.

Model fitness can be quantified utilizing visual predictive check (VPC) plots as shown in Figure 1A and Figure S3 and model goodness-of-fit plots as shown in Figure 1B and Figure S1. The VPC plot showed that the distribution and variability of the observed concentrations was consistent with that of the model-predicted concentrations at the 10th, 50th, and 90th percentiles, supporting model use for simulation studies under dynamic conditions. The observed vs. predicted goodness-of-fit plot showed that the general trend followed the line of identity, indicating that the structural model captured the observed data well.

### 2.3. Hollow-Fiber Infection Model

**Figure 2:**
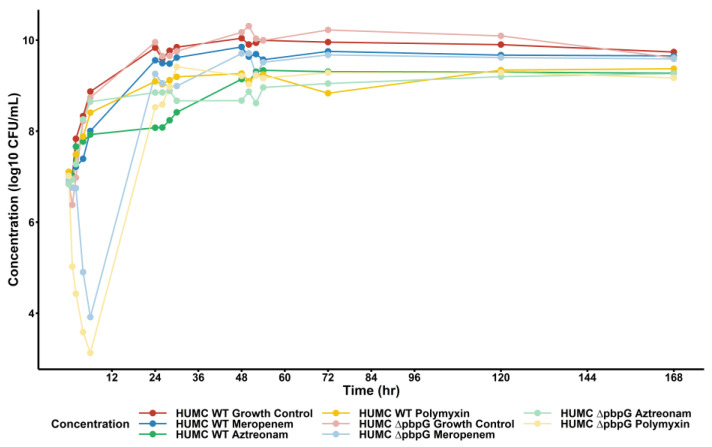
**Hollow-fiber infection model results of *A. baumannii* isolates vs. monotherapy therapies of interest.** Solid points represent observed bacterial concentrations at each HFIM time point. Dark-shaded dots/lines represent the wild-type *A. baumannii* isolate, whereas the light-shaded dots/lines represent the isogenic PBP7/8 knockout strain. For both strains, clinically relevant regimens of meropenem (blue), aztreonam (green), and polymyxin B (yellow) were simulated over 168 h.

The results of the HFIM experiments are summarized in Figure 2 for the total population counts of each tested regimen. Aztreonam demonstrated minimal bacterial killing as expected due to *A. baumannii* being intrinsically resistant. Meropenem and polymyxin B time-kill studies with approximately matching C_max_ were able to prevent 24 h regrowth. However, all therapies tested in the HFIM had concentrations above the starting inoculum by 24 h, reaching an approximate HFIM-carrying capacity of 10^10^ CFU/mL. The population analysis profile (PAP) analysis shown in Figure S4 demonstrates that PAP concentrations displayed appreciable resistance proliferation only when the HFIM antibiotic exposure aligned with the PAP plate. In other cases where HFIM exposure did not match the PAP plate, there was no appreciable resistance proliferation as there was no resistance development driver. The HFIM observations were then retrospectively compared to the model-predicted bacterial killing, demonstrating that our final mechanism-based model was able to appropriately capture changes in bacterial population within a dynamic HFIM scenario.

**Figure 3:**
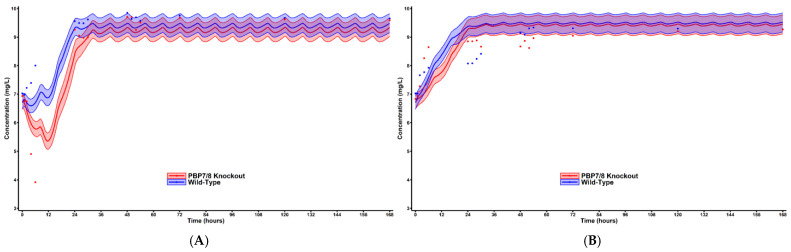
**Simulated bacterial time-course plot for HFIM observations.** Simulated using the pharmacodynamic model estimated from the static concentration conditions of the time-kill experiments with the pharmacokinetic parameters of the HFIM. Solid lines represent the population mean prediction and the 50% prediction intervals. Red represents the wild-type strain while blue represents the PBP7/8 knockout. Simulated dosing performed on **meropenem** (**A**) and **aztreonam** (**B**).

Using the completed mechanism-based model, we prospectively simulated the bacterial time course using the mechanism-based pharmacodynamic model in conjunction with the pharmacokinetic parameters of the hollow-fiber infection model. These simulations were run for hypothetical regimens utilizing meropenem and aztreonam with simulation output shown in Figure 3A,B.

## 3. Discussion

*Acinetobacter baumannii* has become a global urgent health threat. The overprescribing and inappropriate use of antibiotics has also furthered the rise of antimicrobial resistance, making *A. baumannii* invulnerable to many clinically relevant antibiotics [18]. Here, we investigated an orthogonal approach of antibacterial adjuvants to address the dearth of new antibiotic agents.

To accomplish this, we developed a pharmacodynamic evaluation of adjuvant targets (PEAT) approach, which utilized a knockout of the target of interest (PBP7/8) in a clinically relevant isolate. HUMC1ΔPBP7/8 thus served as a surrogate for an “ideal” small molecule adjuvant’s activity against PBP7/8. The first objective was to study the pharmacodynamic effects of PBP7/8 inactivation on a variety of clinically relevant beta-lactams. Static concentration time-kill studies showed that for the HUMC1ΔPBP7/8 knockout isolate, there was a significant increase in sensitivity to each tested antibiotic compared with the wild-type isolate. Treatment efficacy was further enhanced with the utilization of concurrent beta-lactam and polymyxin B exposure. The time-kill studies were performed under the assumption that there would be no significant changes in antibiotic concentration over the timespan of the experiment with the expectation that there is no-to-minimal thermal drug degradation over a 24 h period [19].

The mechanism-based model parameter estimates showed that the presence of PBP7/8 inactivation caused increased susceptibility in the *A. baumannii* isolate, expressed as a negative beta value associated with each antibiotic KC_50_. This decrease in KC_50_ occurred regardless of pre-existing susceptibility or inherent resistance to the antibiotic. Treatment improvements can hypothetically manifest to patients as a possible decrease in dose needed for treatment and as an improvement in safety profile. For example, HUMC1ΔPBP7/8 exhibited lower KC_50_ values of aztreonam and imipenem that were below the *f*C_max_ targets utilized in the static concentration time-kill experiments [20,21,22]. Ultimately, this could lead to a greater duration of time where the in vivo beta-lactam concentration is above its effective concentration, a desirable outcome as beta-lactams operate as time-dependent killing agents [23]. In a general sense, the ability to achieve a *f*C_max_ greater than the KC_50_ value could allow for greater margin of error to accommodate the interindividual pharmacokinetics of a patient or could offer the potential for dose reduction to assuage potential adverse effect concerns while maintaining efficacy. Another observation of interest is that PBP7/8 knockout has synergistic effects with polymyxin B, demonstrating the exploitation of these two distinct mechanisms of increasing bacterial membrane permeability is a viable option, at least in the in vitro setting.

To address our second aim utilizing our PEAT strategy, the completed pharmacodynamic model was utilized to generate simulated bacterial population profiles over time for hypothetical HFIM therapies. This strategy is proposed to demonstrate that parameters calculated by our pharmacodynamic model utilizing static concentration in vitro data can successfully predict bacterial concentration behavior in a dynamic concentration HFIM. Our HFIM shows results that are similar to those of the static time-kills, such that monotherapy exposure results in some cell killing with a following regrowth of bacterial counts within approximately 24 h. This bacterial regrowth and resistance development is also followed with the PAP analysis, which showed the development of the resistant subpopulation at equivalent rate for both WT and HUMC1ΔPBP7/8. This indicates that PBP7/8 inhibition does not appear to delay the expression of antimicrobial resistance mechanisms.

When comparing the simulated bacterial time course to the appropriate HFIM experiment observations, the simulations successfully captured the general decline and recovery of the total bacterial concentration and validated the second aim. One ramification of this strategy is that it offers a method to more quickly and efficiently screen potential small molecule targets in the in vitro setting with quick and relatively less expensive knockout time-kill experiments. Targets with good bactericidal activity can be analyzed with pharmacodynamic modeling before investing resources into molecular screening and synthesis of new chemical entities. Potential small molecule targets that display no or minimal bactericidal activity with knockout testing would be eliminated as candidates earlier in the development period, saving time as well as financial and manpower resources.

This work was limited by the use of identical modeling strategies to describe each antibiotic, despite their slightly different mechanisms of action (i.e., different HMW PBP targets). To allow comparisons between different beta-lactams, we modeled killing for all antibiotics utilizing a Hill-type function which represented saturable killing, a common feature among antibacterials. For polymyxins, however, there are published mechanism-based studies suggesting that a non-saturable second-order killing process is more accurate [24]. We also further simplified the modeling strategy by omitting other previously reported biological effects into the final model [24,25]. As the main purpose of this study was to focus more on our hypothesis that PBP7/8-knockout-induced permeabilization would synergize with other beta-lactams, the addition of these model features would not appreciably contribute to demonstrating the validity of this hypothesis. Additionally, our initial time-kill studies were performed in a static in vitro environment without the dynamic administration of antibiotics and grown in rich media. By comparison, an in vivo infection setting would further benefit from synergy with the host’s immune system. With this context, the change in KC_50_ values supports the potential utility of PBP7/8 inactivation as a method to improve the efficacy of existing antimicrobials.

## 4. Materials and Methods

### 4.1. Bacterial Isolates and Media

All experiments were conducted using two isogenic clinical isolates of *Acinetobacter baumannii* HUMC1 (WT) (MIC_Polymyxin_ = 2 mg/L, MIC_Meropenem_ = 32 mg/L) and HUMC1ΔPBP7/8) [26]. The lack of off-target effects or unintended mutation was confirmed with the use of the complemented derivative HUMC1ΔpbpG/pNLAC1[pbpG], which confirmed that the observed phenotypic differences between HUMC1 and HUMC1ΔPBP7/8 were due to PBP7/8 [16]. Fresh bacteria cultures were grown at 37 °C 24 h just prior to all experiments using cation-adjusted Mueller–Hinton broth (BD Difco, Sparks, MD, USA) cation-adjusted with Mg^2+^ (12.5 mg/L) and Ca^2+^ (25 mg/L). Bacterial suspensions were washed prior to use to remove extracellular debris, including any beta-lactamases present in the extracellular matrix. Washing was performed by centrifugation at 5000 rcf for 10 min, decanting the supernatant, resuspending in fresh MHB, then repeating the wash for a total of 4 times. The final wash was resuspended in sterile normal saline and diluted to achieve a starting inoculum of 10^7^ CFU/mL.

### 4.2. Static Concentration Time-Kill Experiments

Fresh stocks of antibiotics were prepared on the day of each experiment. Monotherapies of meropenem (AK Scientific, Union City, CA, USA), imipenem (AK Scientific, Union City, CA, USA), aztreonam (Sigma-Aldrich, St. Louis, MO, USA), ceftazidime/avibactam (Sigma-Aldrich, St. Louis, MO, USA, AK Scientific, Union City, CA, USA), and polymyxin B (Sigma-Aldrich, St. Louis, MO, USA) were utilized. Additionally, combination therapy with each beta-lactam and polymyxin B was explored as a comparator of permeability-enhancing effects. Experiments were conducted using concentrations of meropenem 25, 50, and 100 mg/L, imipenem 7.7, 15.3, and 30.6 mg/L, aztreonam 33, 66, and 132 mg/L, ceftazidime/avibactam 37/5, 73/9.9, and 146/19.8 mg/L, and polymyxin B 0.75, 1.5, 3, and 6 mg/L. Concentrations for each beta-lactam were selected based on the predicted 0.5×, 1×, and 2× free maximum concentrations (fC_max_) determined from Monte Carlo simulations of previous population pharmacokinetic studies. Meropenem concentration was based on a 1 g 30 min infusion Q8H regimen [27], imipenem concentration was based on a 1 g 60 min infusion Q8H regimen [22], aztreonam concentration was based on a 2 g 2 h prolonged infusion Q8H regimen [20,21], and ceftazidime/avibactam was based on a 2 g/0.5 g 2 h prolonged infusion Q8H regimen [21,28]. Polymyxin B concentrations were based on predicted 0.5×, 1×, 2×, and 4× free steady-state average concentrations (fC_ss,avg_), corresponding to guideline target concentrations. Combination therapies included a beta-lactam antibiotic with the addition of polymyxin B using the full concentrations array as outlined above. After sample dilution, all experiments were incubated under constant shaking at 30RPM at 37 °C and samples were collected at 0, 1, 2, 4, 6, 8, and 24 h. Samples were serially diluted in saline, plated on Mueller–Hinton agar (BD Difco, Sparks, MD, USA), and then incubated for 24 h at 37 °C. Colony counts were enumerated utilizing a Protos 3 automated colony counter (Symbiosis, Cambridge UK). The experimental limit of detection was 10^2^ CFU/mL. All experiments were performed in duplicate at minimum using the same experimental design on separate days.

### 4.3. Mechanism-Based Mathematical Model

The data were modeled using nonlinear mixed effects modeling utilizing Monolix software (2023R1, Antony, France) with model equations as described in Figure S5. Data were modeled as log_10_-transformed values. The initial conditions are described as follows. Samples below the quantification limit were modeled as censored data records. Bacterial replication was described using a life-cycle growth model with a subpopulation-based model structure, consisting of a majority subpopulation of susceptible cells, and the remainder being antibiotic-resistant cells [29]. Each subpopulation was modeled either in a “growing” phase or a rapid replicative phase. Bacterial killing was assumed to follow a Hill-type function with a different maximum rate of bacterial killing for the antibiotic-susceptible and antibiotic-resistant bacterial populations.

To model PBP7/8 synergy, changes in beta-lactam EC_50_ via PBP7/8 knockout were statistically tested for as a covariate effect. All time-kill data for the two *A. baumannii* strains were modeled simultaneously based on the total population concentration counts of each isolate. Interexperimental variability was acknowledged by assuming a coefficient of variation of 5% for each model parameter. Residual variability was modeled as additive, such that its use with log_10_-transformed values translates to proportional error with non-transformed values.

### 4.4. Prospective Simulation of PBP7/8 Inactivation Effects

To simulate human antibiotic dosing, three different antibiotic regimens were administered in the HFIM. The following dosing schemes were simulated with beta-lactams administered as a 3 h prolonged infusion and polymyxin as a bolus infusion. The regimens were structured to achieve approximately the free C_max_ of clinical interest as mentioned above in the static concentration time-kill experiments. The regimens are described as below:

Untreated, Growth Control

Meropenem 2 g Q8H infused over 3 h, (fC_max_ = 54.3 mg/L)

Aztreonam 2 g Q8H infused over 3 h, (fC_max_ = 63.7 mg/L)

Polymyxin B 2.22 mg/kg × 1 bolus, then 1.43 mg/kg Q12H, (fC_max_ = 2.41 mg/L)

### 4.5. Hollow-Fiber Infection Model Experiments

A hollow-fiber infection model (HFIM) was utilized to quantify beta-lactam pharmacodynamics in HUMC1 and HUMC1ΔPBP7/8 towards antimicrobial regimens mimicking physiological conditions [30]. Cellulosic cartridges (C8008, FiberCell Systems, Frederick, MD, USA) were used for all HFIM experiments. Fresh cation-adjusted Mueller–Hinton broth was infused into a central reservoir at a rate of 0.8 mL/min. Fresh antibiotic solution was also infused into the central reservoir, with the reservoir outflow set to mimic a drug half-life of 130 min in order to mimic the reduced renal and hepatic function of a critically ill patient [31]. A starting inoculum was introduced into the extracapillary space of each cartridge to achieve a starting concentration of 10^7^ CFU/mL. All HFIM experiments had sample collection at 0, 1, 2, 4, 6, 24, 26, 28, 30, 48, 50, 52, 54, 72, 120, and 168 h. Total bacterial concentration at each time point was quantified via plating on Mueller–Hinton agar using the same methods as described above. Population analysis profiles (PAPs) to track the resistant subpopulation over the length of the experiment were measured using MHA plates containing 0, 1, 4, and 16 mg/L meropenem, 0, 4, 16, and 64 mg/L aztreonam, or 0, 1, 4, and 16 polymyxin B to profile the resistant subpopulation. PAP cultures were incubated for 48 h before counting utilizing the same methods as described above.

## 5. Conclusions

In conclusion, the emergence and prevalence of drug-resistant *A. baumannii* has precipitated a need for novel targets. By using our PEAT strategy to evaluate PBP7/8 as a possible antibacterial adjuvant, we have shown that its inactivation resulted in the sensitization of our *A. baumannii* isolate to a variety of clinically relevant beta-lactam antibiotics. Our results suggest that the development of a PBP7/8-targeting adjuvant could offer great public health benefits by potentiating previously ineffective CRAB therapies or by decreasing the dose of antibiotics required for successful treatment. Further work to identify small molecule compounds specifically capable of inhibiting PBP7/8 activity for next-stage studies would be an ideal next step. Additionally, the in vitro findings in this study should be translated into an in vivo environment such as a murine or rabbit model to further explore the effectiveness of PBP7/8-inhibiting-adjuvant activity in a host capable of mounting an immune response. Lastly, although this study focuses on the treatment of CRAB due to its position as an urgent global health threat, the results can be extended to other Gram-negative species that are similarly challenging to treat due to the development of resistance mechanisms.

## Figures and Tables

**Table 1:** Parameter estimates obtained from the final mechanism-based model used to describe the static concentration time-kill data.

Parameter	Description	Unit	Value (RSE%)
LOGCFU_max_	Maximum carrying capacity concentration	Log_10_CFU/mL	8.20 (0.91%)
LOGINOC	Starting inoculum concentration	Log_10_CFU/mL	6.71 (0.38%)
LOGMUTR	Population mutation frequency	-	5.03 (0.21%)
MGT_S_	Mean growth time of susceptible (S) subpopulation	min	75.3 (0.51%)
MGT_R_	Mean growth time of resistant (R) subpopulation	min	46.4 (1.06%)
K_max,S,MER_	Maximal killing of (S) subpopulation by meropenem	h^−1^	2.09 (2.96%)
K_max,S,IMI_	Maximal killing of (S) subpopulation by imipenem	h^−1^	1.56 (2.54%)
K_max,S,ATM_	Maximal killing of (S) subpopulation by aztreonam	h^−1^	1.12 (0.98%)
K_max,S,CEF_	Maximal killing of (S) subpopulation by ceftazidime/avibactam	h^−1^	1.52 (3.12%)
K_max,S,PMB_	Maximal killing of (S) subpopulation by polymyxin B	h^−1^	2.98 (1.11%)
K_max,R,MER_	Maximal killing of (R) subpopulation by meropenem	h^−1^	1.13 (2.01%)
K_max,R,IMI_	Maximal killing of (R) subpopulation by imipenem	h^−1^	0.83 (1.90%)
K_max,R,ATM_	Maximal killing of (R) subpopulation by aztreonam	h^−1^	0.76 (1.60%)
K_max,R,CEF_	Maximal killing of (R) subpopulation by ceftazidime/avibactam	h^−1^	0.89 (1.97%)
K_max,R,PMB_	Maximal killing of (R) subpopulation by polymyxin B	h^−1^	1.12 (0.84%)
KC_50,MER_	Meropenem concentration for 50% of maximal killing of population	mg/L	47.6 (9.42%)
KC_50,IMI_	Imipenem concentration for 50% of maximal killing of population	mg/L	16.8 (10.6%)
KC_50,ATM_	Aztreonam concentration for 50% of maximal killing of population	mg/L	78.6 (14.4%)
KC_50,CEF_	Ceftazidime/avibactam concentration for 50% of maximal killing of population	mg/L	76.0 (10.8%)
KC_50,PMB_	Polymyxin B concentration for 50% of maximal killing of population	mg/L	1.00 (0.34%)
β_KC50,MER_	Effect of PBP7/8 knockout on KC_50_ for meropenem	-	−0.80 (13.0%)
	Calculated KC_50_ of meropenem in PBP7/8 knockout	mg/L	21.4
β_KC50,IMI_	Effect of PBP7/8 knockout on KC_50_ for imipenem	-	−1.10 (10.4%)
	Calculated KC_50_ of imipenem in PBP7/8 knockout	mg/L	6.6
β_KC50,ATM_	Effect of PBP7/8 knockout on KC_50_ for aztreonam	-	−1.02 (19.1%)
	Calculated KC_50_ of aztreonam in PBP7/8 knockout	mg/L	28.3
β_KC50,CEF_	Effect of PBP7/8 knockout on KC_50_ for ceftazidime/avibactam	-	−0.89 (17.2%)
	Calculated KC_50_ of ceftazidime/avibactam in PBP7/8 knockout	mg/L	31.2
β_KC50,PMB_	Effect of PBP7/8 knockout on KC_50_ for polymyxin B	-	−0.50 (5.25%)
	Calculated KC_50_ of polymyxin B in PBP7/8 knockout	mg/L	0.61
Hill_MER_	Shape parameter on meropenem killing	-	0.91 (1.46%)
Hill_IMI_	Shape parameter on imipenem killing	-	0.88 (1.19%)
Hill_ATM_	Shape parameter on aztreonam killing	-	0.51 (4.08%)
Hill_CER_	Shape parameter on ceftazidime/avibactam killing	-	0.90 (1.99%)
Hill_PMB_	Shape parameter on polymyxin B killing	-	0.93 (0.35%)

## Data Availability

The original contributions presented in the study are included in the article/Supplementary Materials. Further inquiries can be directed to the corresponding authors.

## Supplementary Materials

The following supporting information can be downloaded at: https://www.mdpi.com/article/10.3390/ph18060918/s1, Figure S1. Observed vs. predicted value goodness-of-fit plots stratified by isolate and antibiotic therapy. Figure S2. Population predicted vs. population weighted residual goodness-of-fit plots stratified by isolate and antibiotic therapy. Figure S3. Visual predictive check plots stratified by isolate and antibiotic therapy. Figure S4. Population analysis profile plots for aztreonam, meropenem, and polymyxin B regimens explored in the HFIM; Figure S5. Model Equations.
