# Supplementary material for: Pharmacodynamic Evaluation of Adjuvant Targets: Low Molecular Weight PBP7/8 Effects on β-Lactam Activity Against Carbapenem-Resistant Acinetobacter Baumannii
Source: Pharmaceuticals (Basel). 2025 Jun 18;18(6):918. doi: 10.3390/ph18060918 (PMC12196172; doi:10.3390/ph18060918)
Supplement: Supplementary file 1 [file pharmaceuticals-18-00918-s001.zip › pharmaceuticals-3680971-supplementary.pdf]

SUPPLEMENTAL MATERIALS

Quantifying and Predicting the Effects of Low Molecular Weight PBP7/8 Knockout on Beta-lactam Pharmacodynamics in Carbapenem-resistant *Acinetobacter baumannii*

Brian M. Ho[1], Jingxiu Jin[1], Jacob Sanborn[1], Thomas D. Nguyen[1], Navaldeep Singh[1], Christina Cheng[1], Nader N. Nasief[1], Ulrike Carlino-MacDonald[2,3], Thomas A. Russo[2,3,4], Nicholas M. Smith[1,*]

[1]Department of Pharmacy Practice, School of Pharmacy and Pharmaceutical Sciences, University at Buffalo, The State University of New York, Buffalo, New York, USA; [2]Department of Medicine, Jacobs School of Medicine and Biomedical Sciences, University at Buffalo, The State University of New York, Buffalo, New York, USA. [3]Veterans Administration Western New York Healthcare System, Buffalo, New York, USA; [4]The Witebsky Center for Microbial Pathogenesis, University at Buffalo, The State University of New York, Buffalo, New York, USA.

A

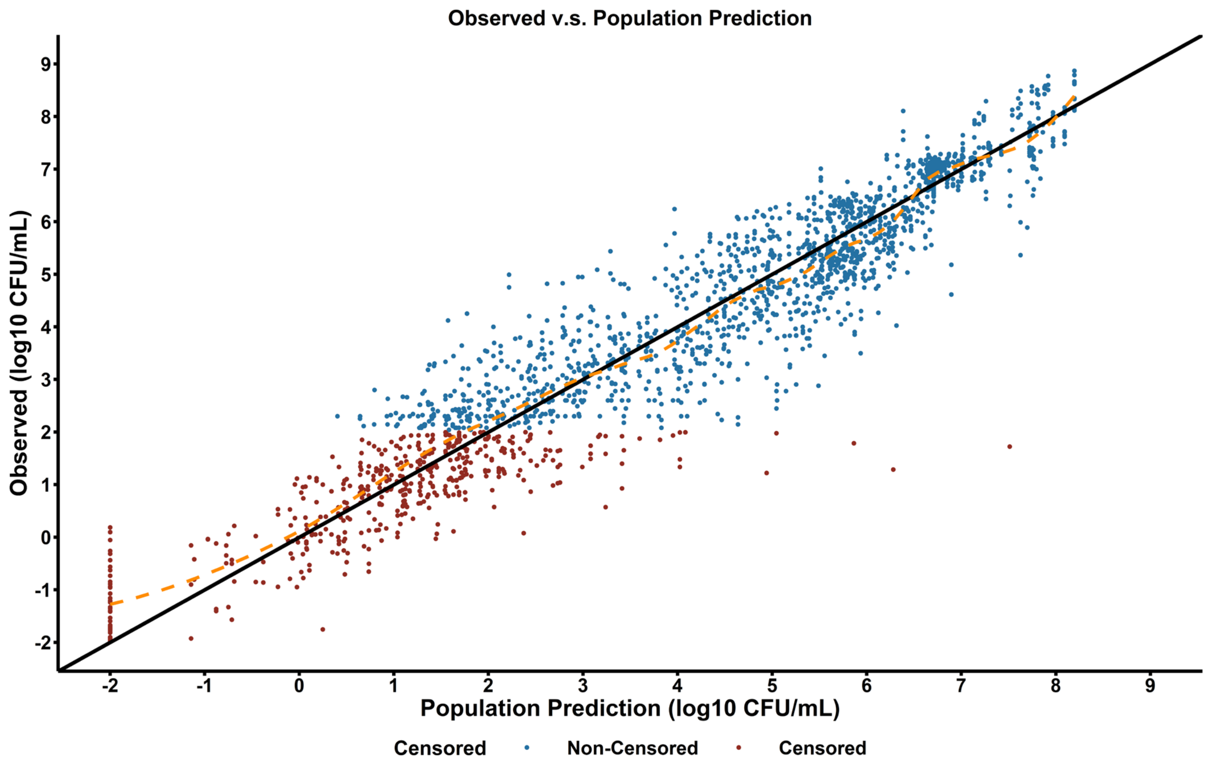

Observed v.s. Population Prediction
Observed (log10 CFU/mL)
Population Prediction (log10 CFU/mL)
-2
-1
0
1
2
3
4
5
6
7
8
9
Censored
Non-Censored
Censored

B

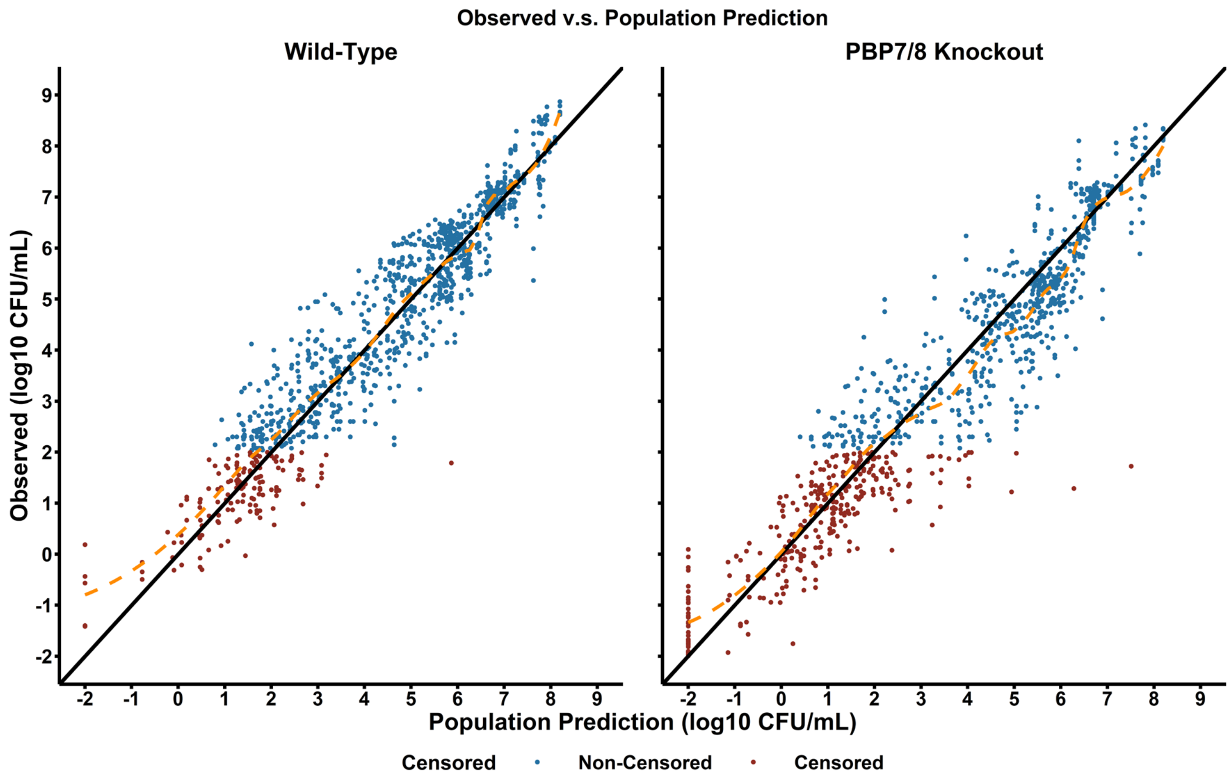

Observed v.s. Population Prediction
Wild-Type
PBP7/8 Knockout
Observed (log10 CFU/mL)
Population Prediction (log10 CFU/mL)
-2
-1
0
1
2
3
4
5
6
7
8
9
Censored
Non-Censored
Censored

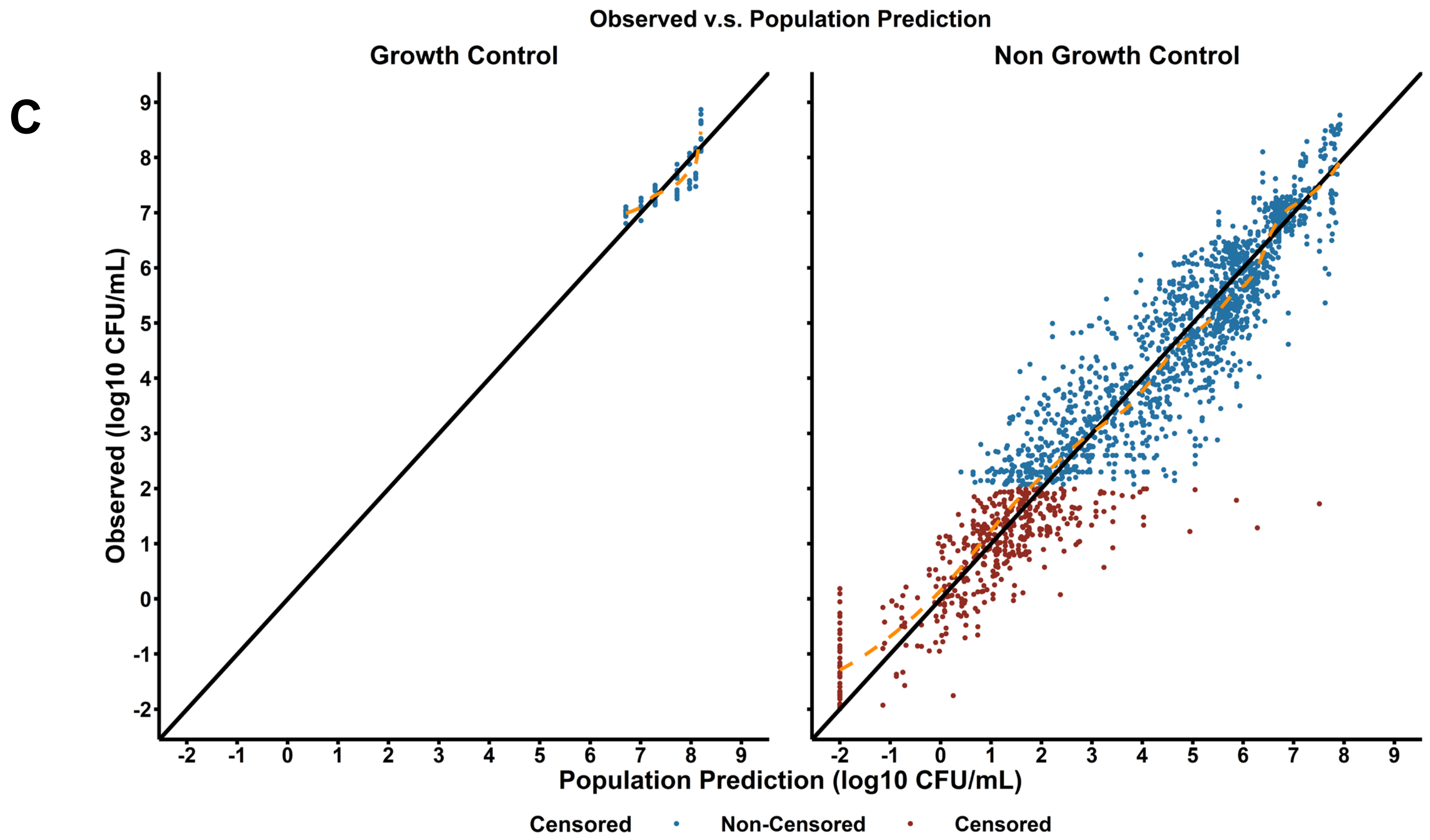
C
Observed v.s. Population Prediction
Growth Control
Non Growth Control
Observed (log10 CFU/mL)
Population Prediction (log10 CFU/mL)
Censored
Non-Censored
Censored

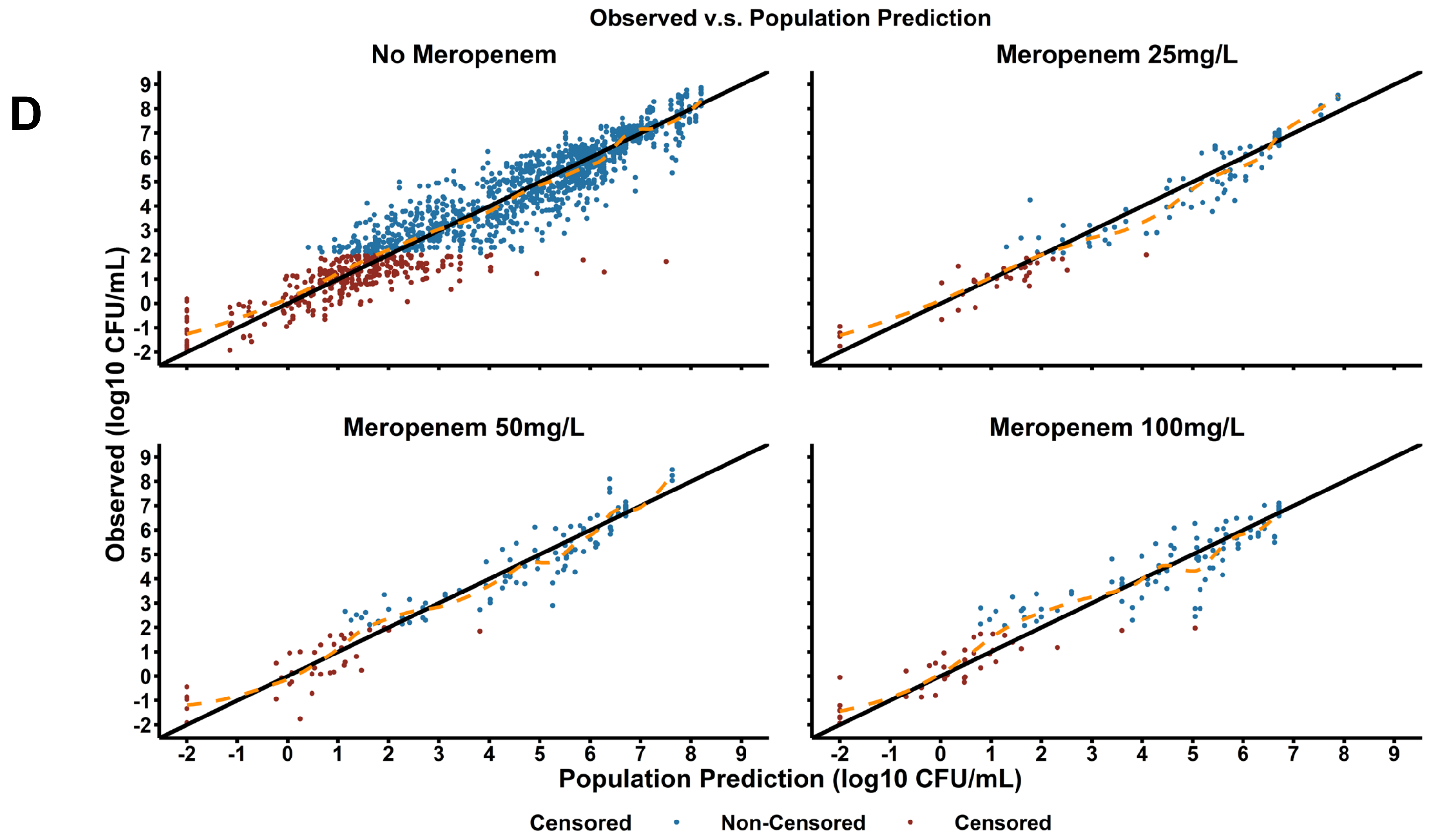
D
Observed v.s. Population Prediction
No Meropenem
Meropenem 25mg/L
Meropenem 50mg/L
Meropenem 100mg/L
Observed (log10 CFU/mL)
Population Prediction (log10 CFU/mL)
Censored
Non-Censored
Censored

E

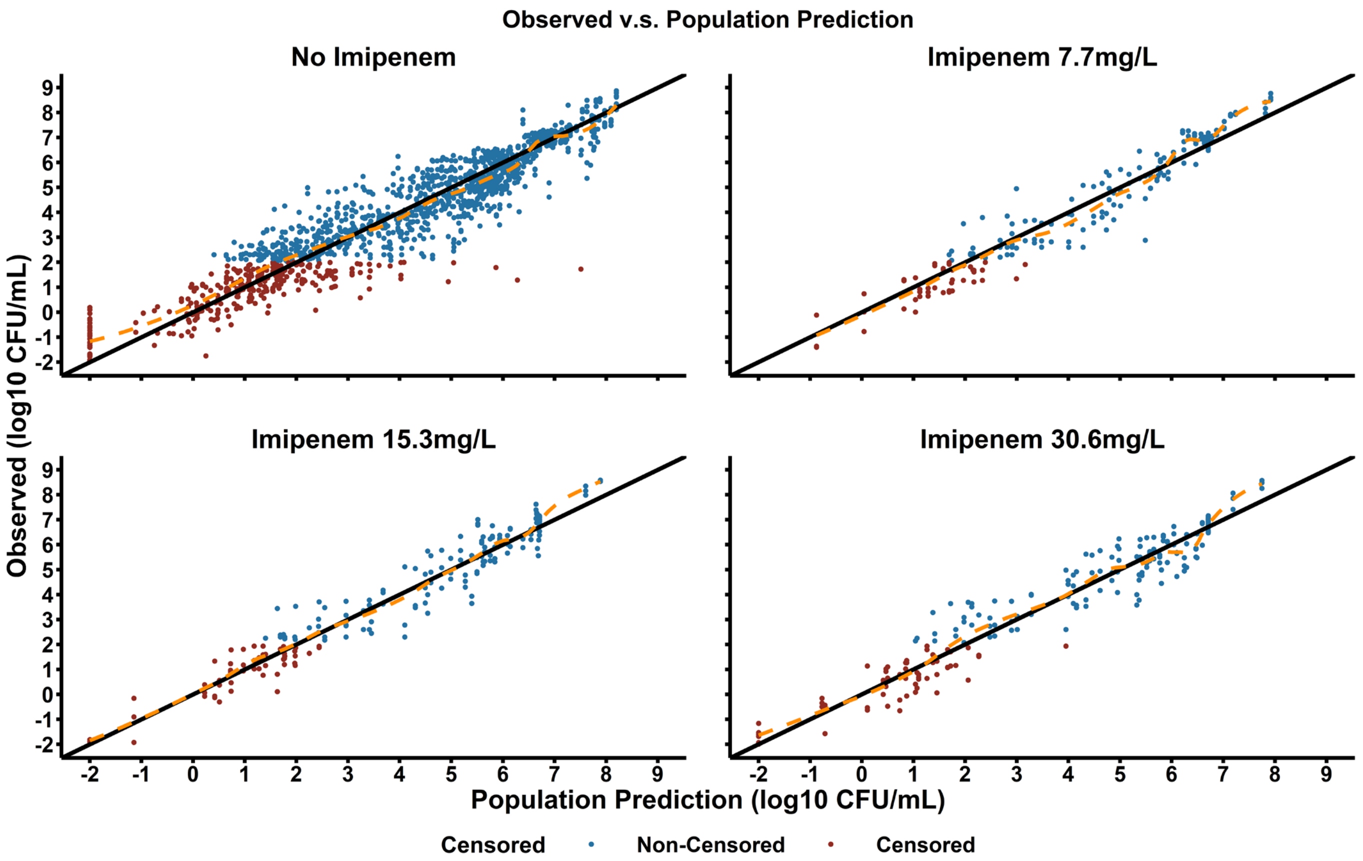
Observed v.s. Population Prediction
No Imipenem
Imipenem 7.7mg/L
Imipenem 15.3mg/L
Imipenem 30.6mg/L
Observed (log10 CFU/mL)
Population Prediction (log10 CFU/mL)
Censored
Non-Censored
Censored

F

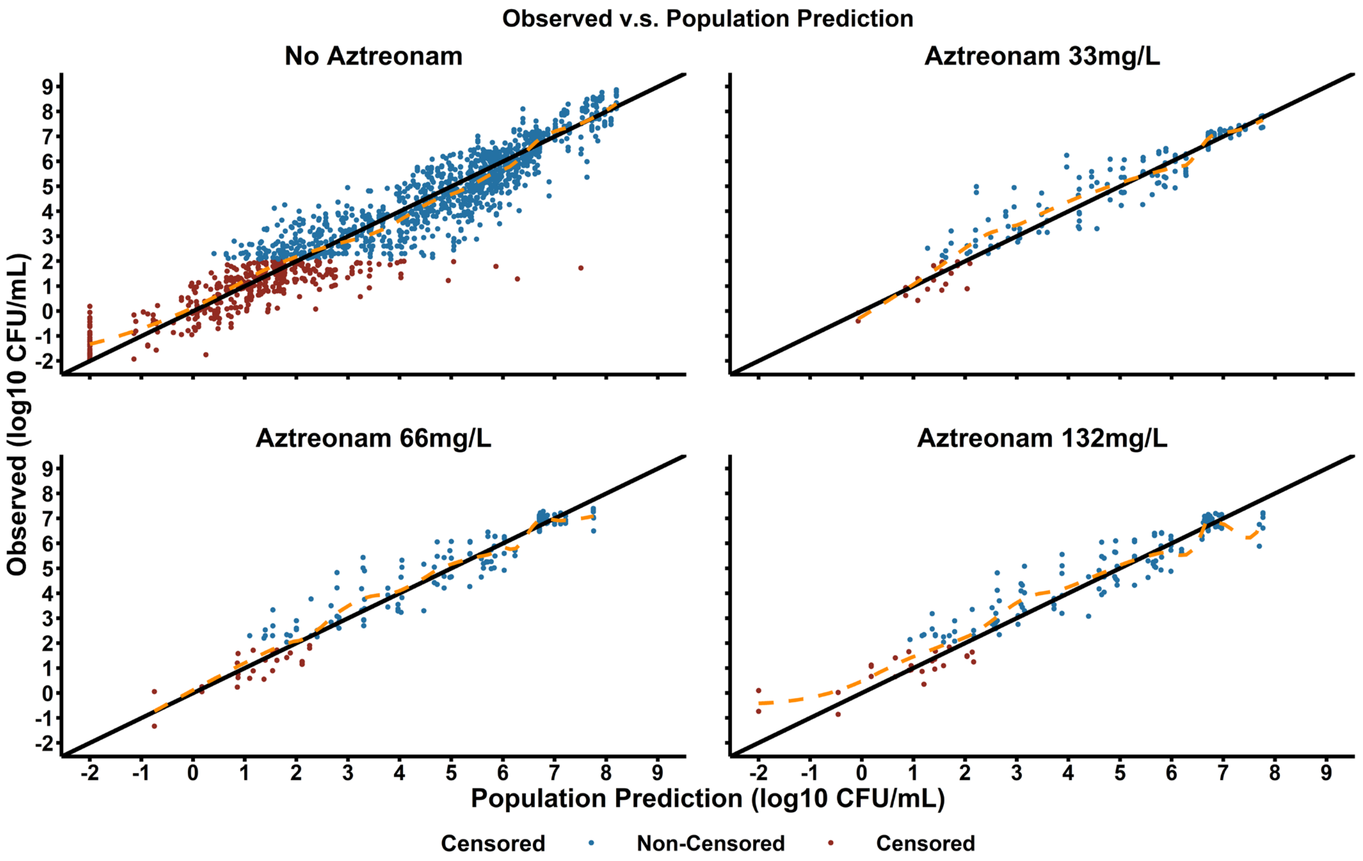
Observed v.s. Population Prediction
No Aztreonam
Aztreonam 33mg/L
Aztreonam 66mg/L
Aztreonam 132mg/L
Observed (log10 CFU/mL)
Population Prediction (log10 CFU/mL)
Censored
Non-Censored
Censored

G

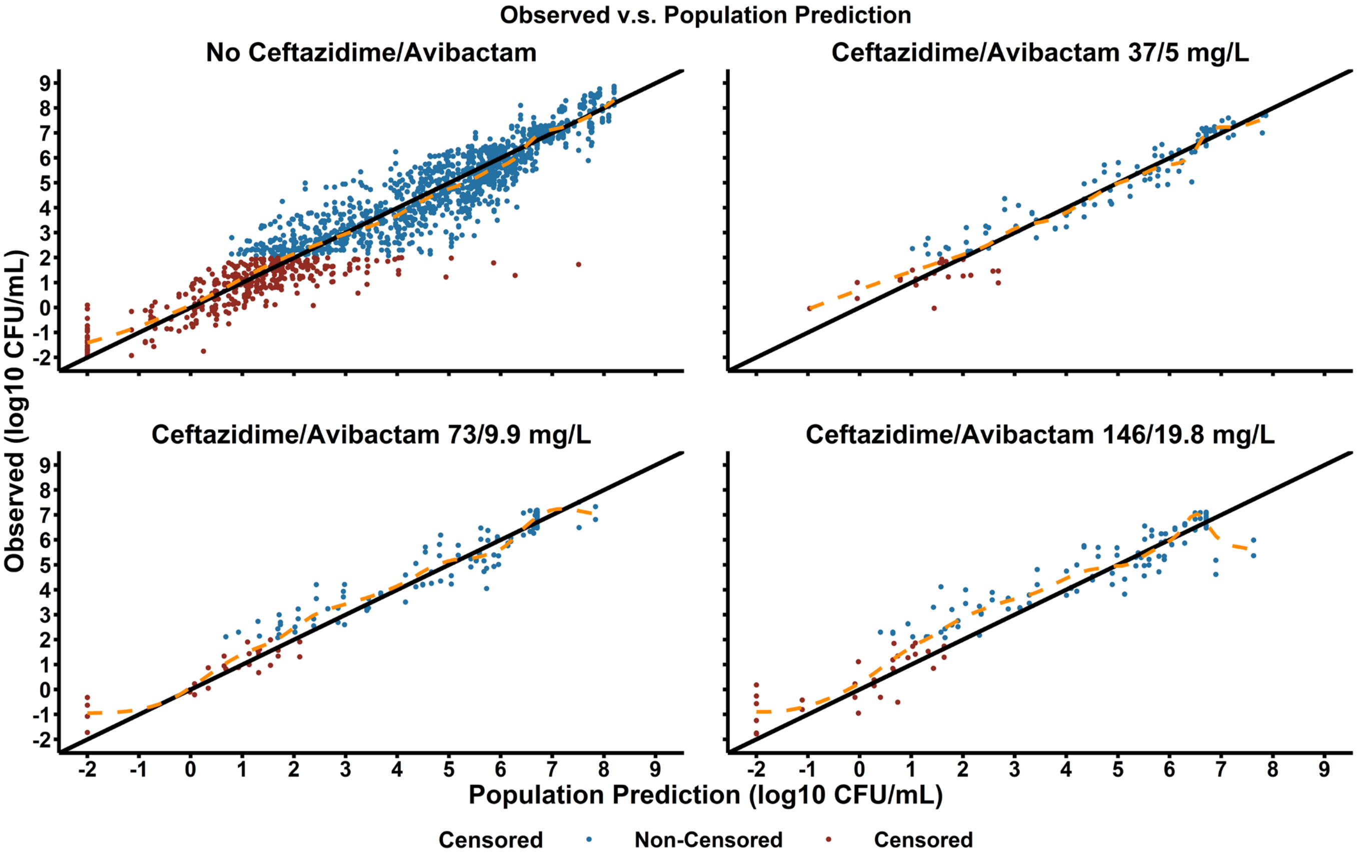

Observed v.s. Population Prediction
No Ceftazidime/Avibactam
Ceftazidime/Avibactam 37/5 mg/L
Ceftazidime/Avibactam 73/9.9 mg/L
Ceftazidime/Avibactam 146/19.8 mg/L
Observed (log10 CFU/mL)
Population Prediction (log10 CFU/mL)
Censored
Non-Censored
Censored

H

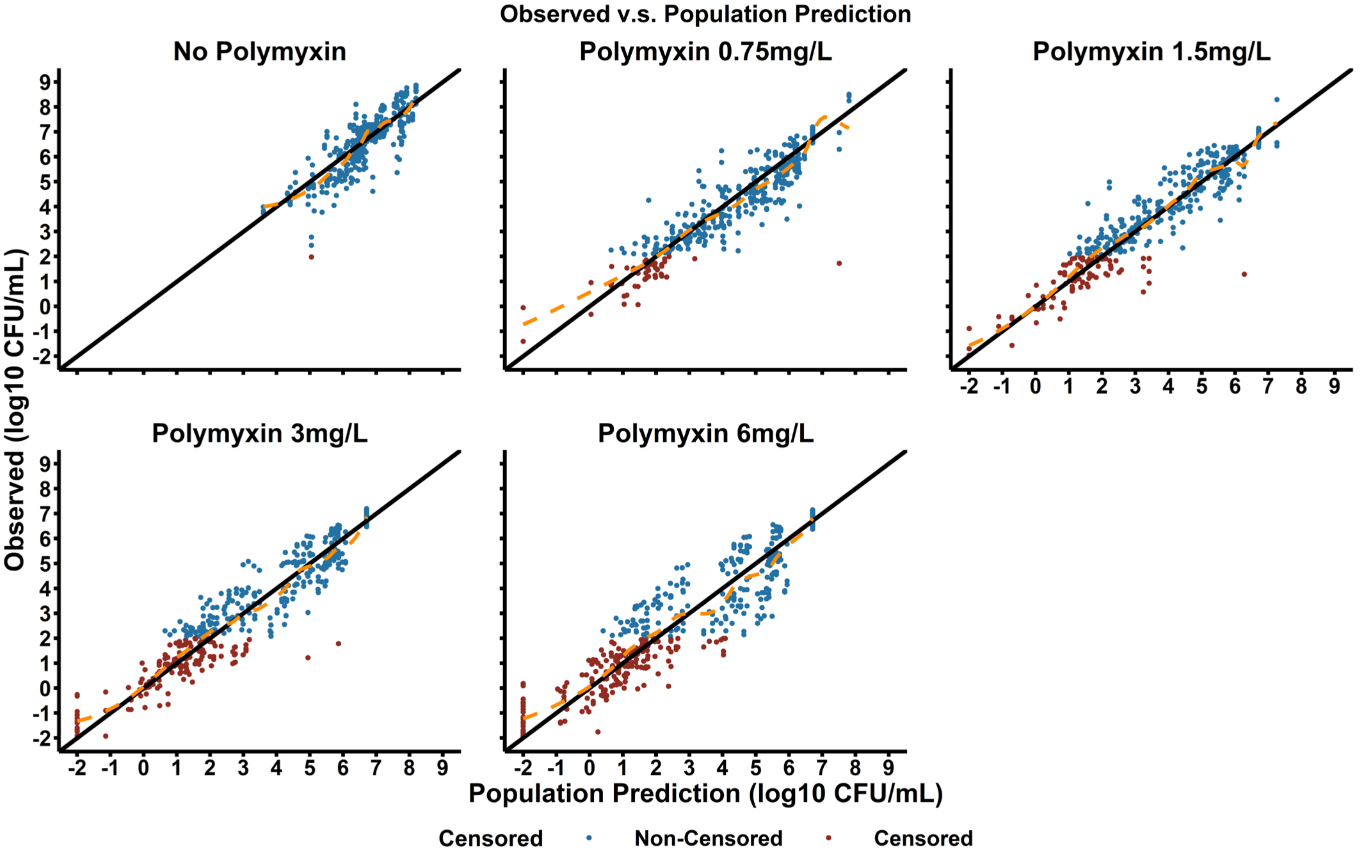

Observed v.s. Population Prediction
No Polymyxin
Polymyxin 0.75mg/L
Polymyxin 1.5mg/L
Polymyxin 3mg/L
Polymyxin 6mg/L
Observed (log10 CFU/mL)
Population Prediction (log10 CFU/mL)
Censored
Non-Censored
Censored

Figure S1. Observed versus Predicted Value Plots. Each plot indicates observed versus predicted values of total population (A), PBP7/8 knockout covariate (B), and for each monotherapy (C-H). Solid line represents the line of identity and dashed line represents the spline line indicating the general trend of data.

A

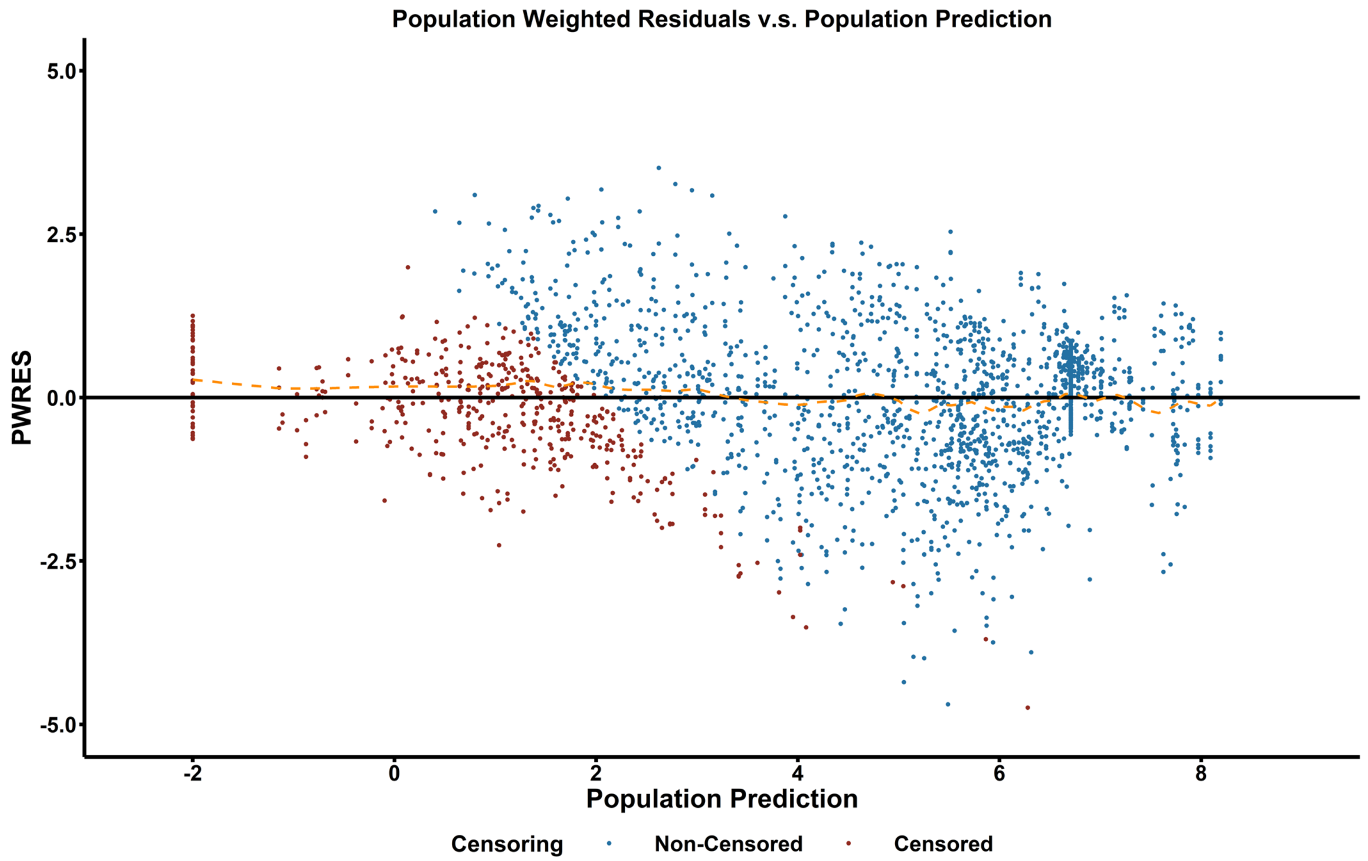

B

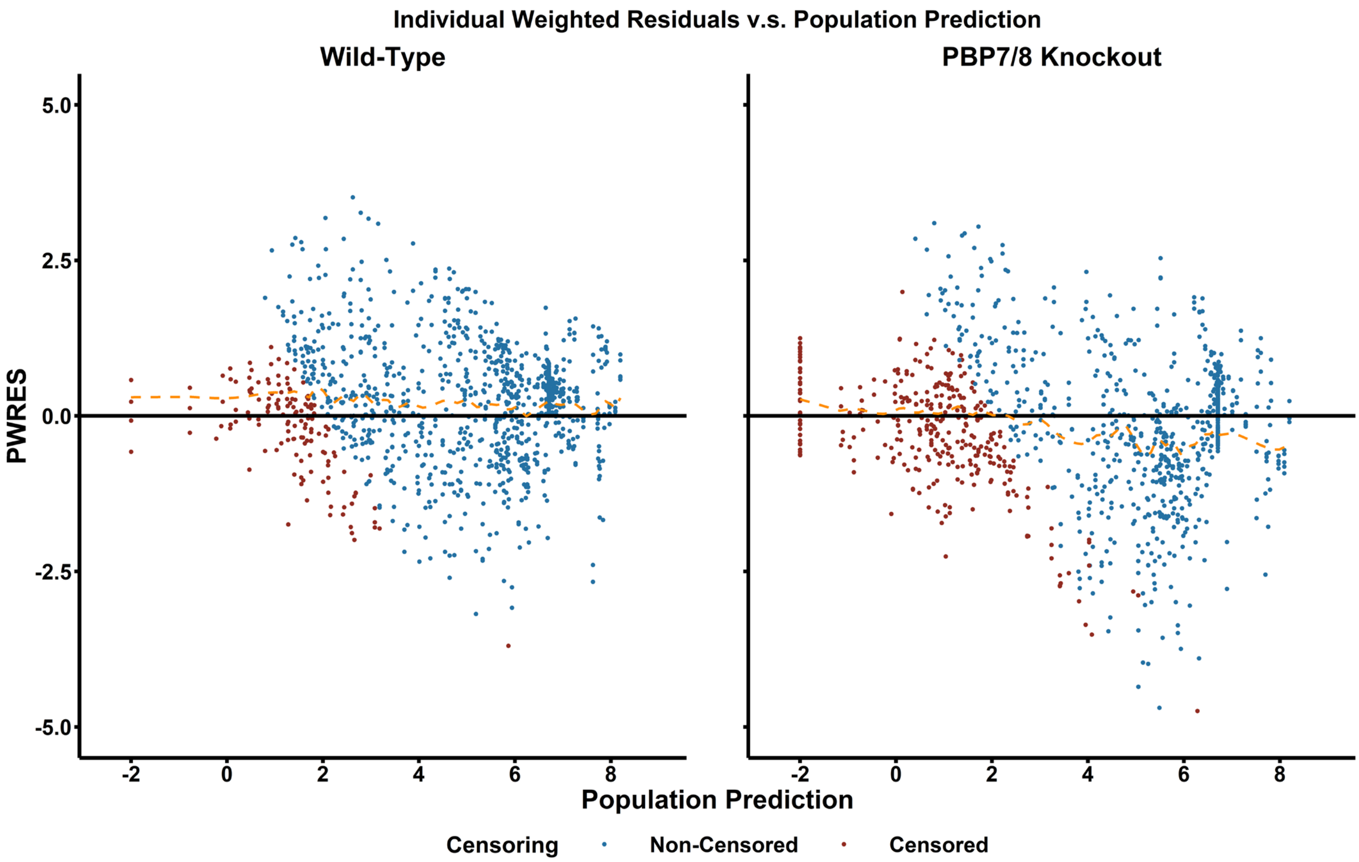
Individual Weighted Residuals v.s. Population Prediction
Wild-Type
PBP7/8 Knockout
PWRES
5.0
2.5
0.0
-2.5
-5.0
-2
0
2
4
6
8
Population Prediction
Censoring
Non-Censored
Censored

C

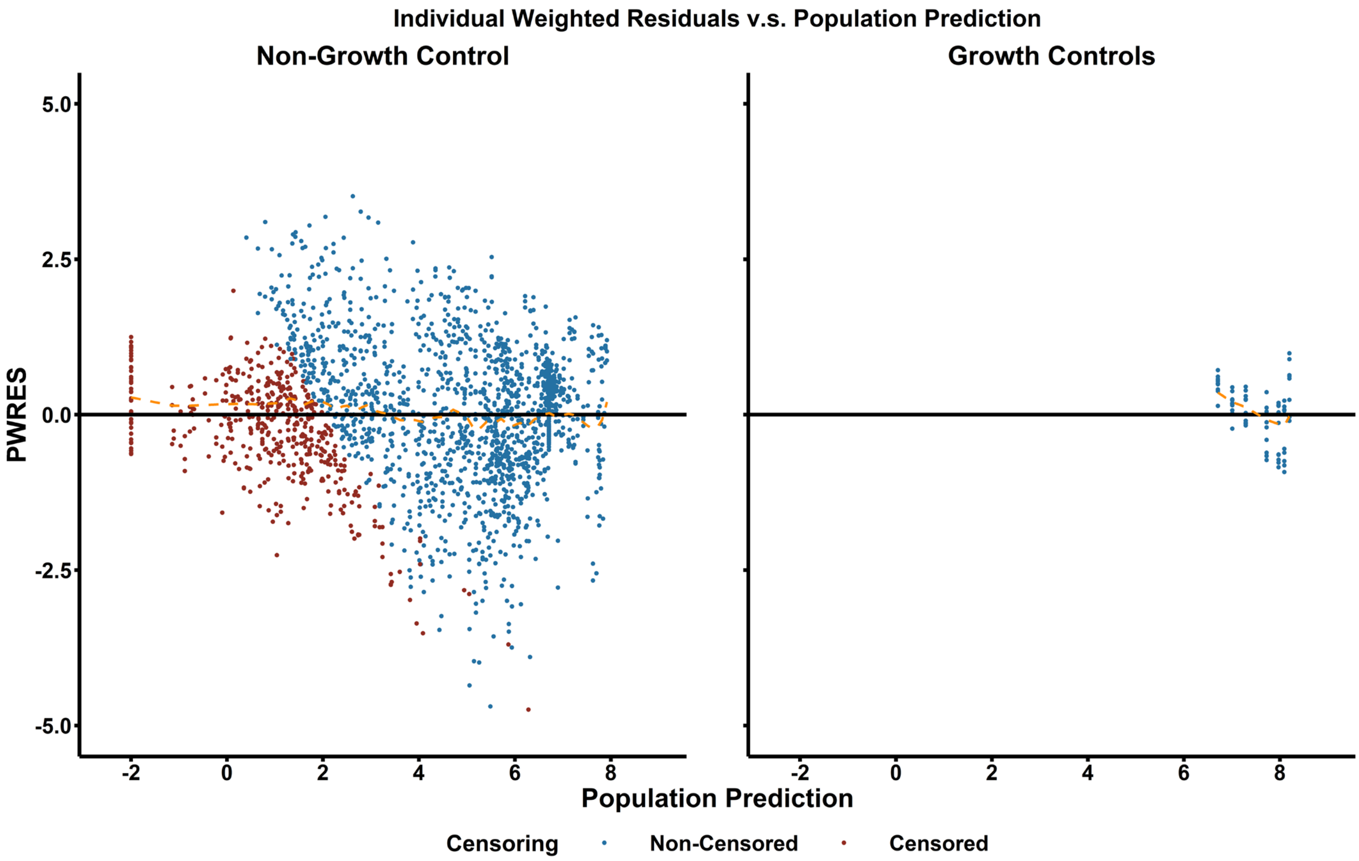
Individual Weighted Residuals v.s. Population Prediction
Non-Growth Control
Growth Controls
PWRES
5.0
2.5
0.0
-2.5
-5.0
-2
0
2
4
6
8
Population Prediction
Censoring
Non-Censored
Censored

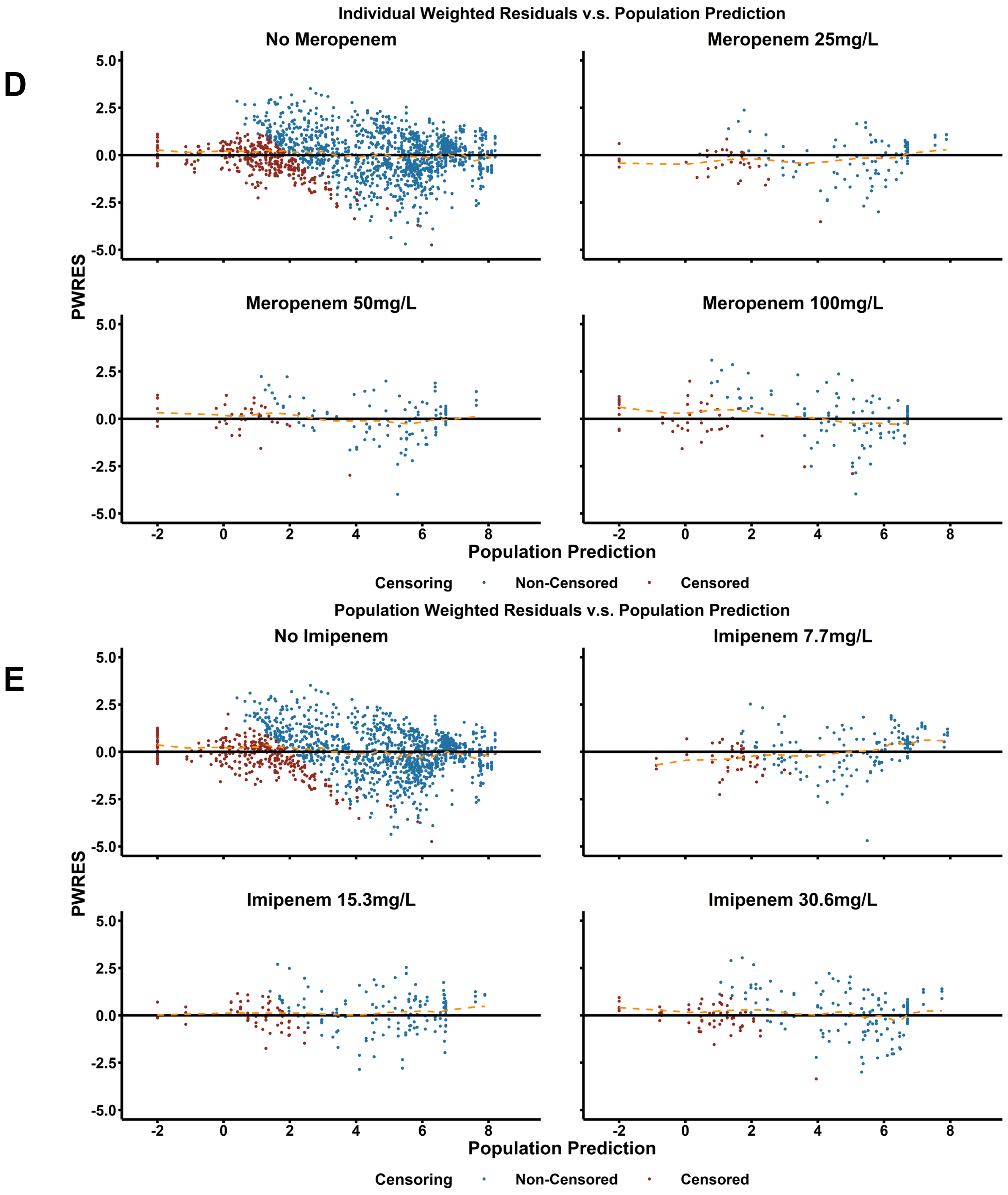
Individual Weighted Residuals v.s. Population Prediction
D
No Meropenem
Meropenem 25mg/L
Meropenem 50mg/L
Meropenem 100mg/L
PWRES
5.0
2.5
0.0
-2.5
-5.0
-2
0
2
4
6
8
Population Prediction
Censoring
Non-Censored
Censored
Population Weighted Residuals v.s. Population Prediction
E
No Imipenem
Imipenem 7.7mg/L
Imipenem 15.3mg/L
Imipenem 30.6mg/L

F

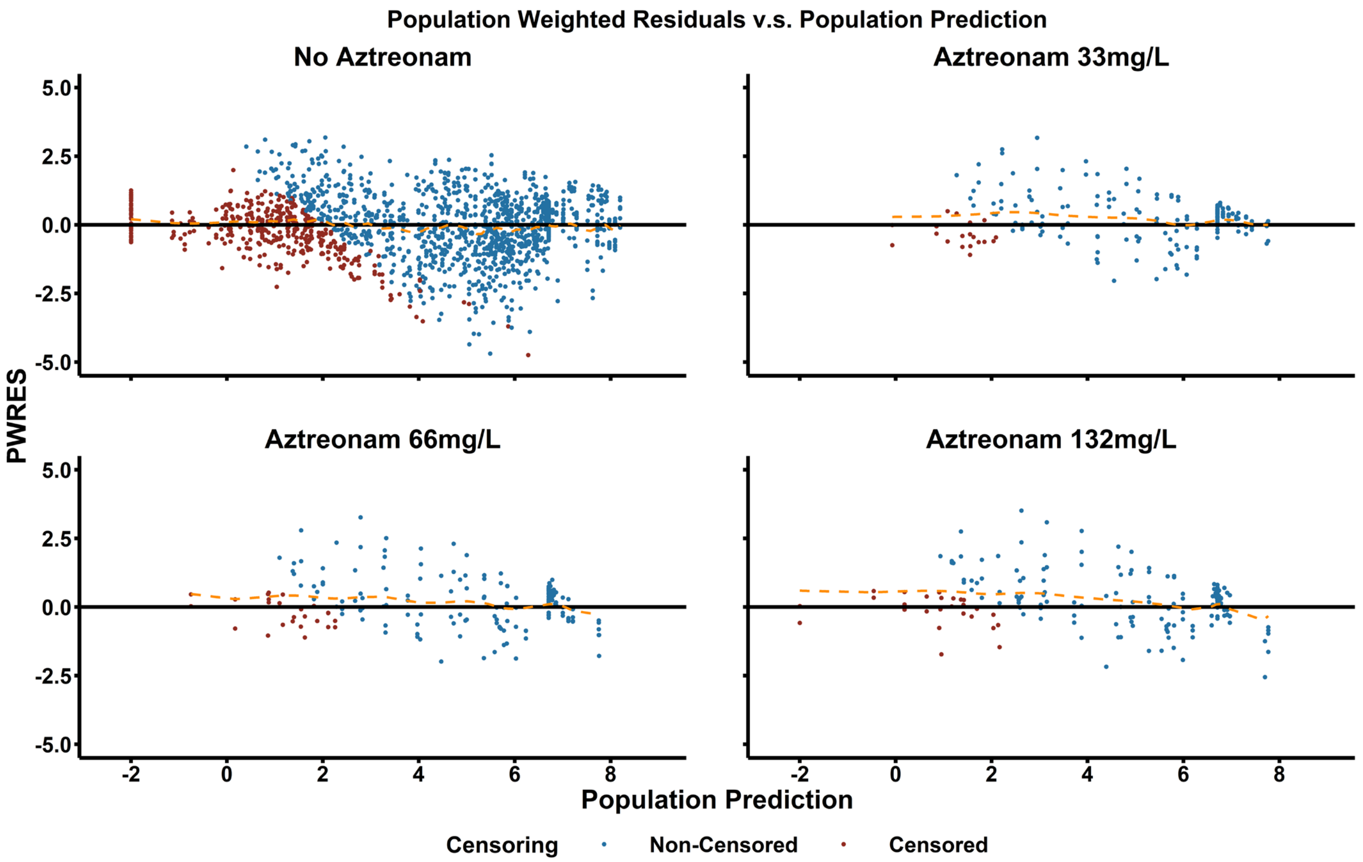

Population Weighted Residuals v.s. Population Prediction
No Aztreonam
Aztreonam 33mg/L
Aztreonam 66mg/L
Aztreonam 132mg/L
PWRES
5.0
2.5
0.0
-2.5
-5.0
-2
0
2
4
6
8
Population Prediction
Censoring
Non-Censored
Censored

G

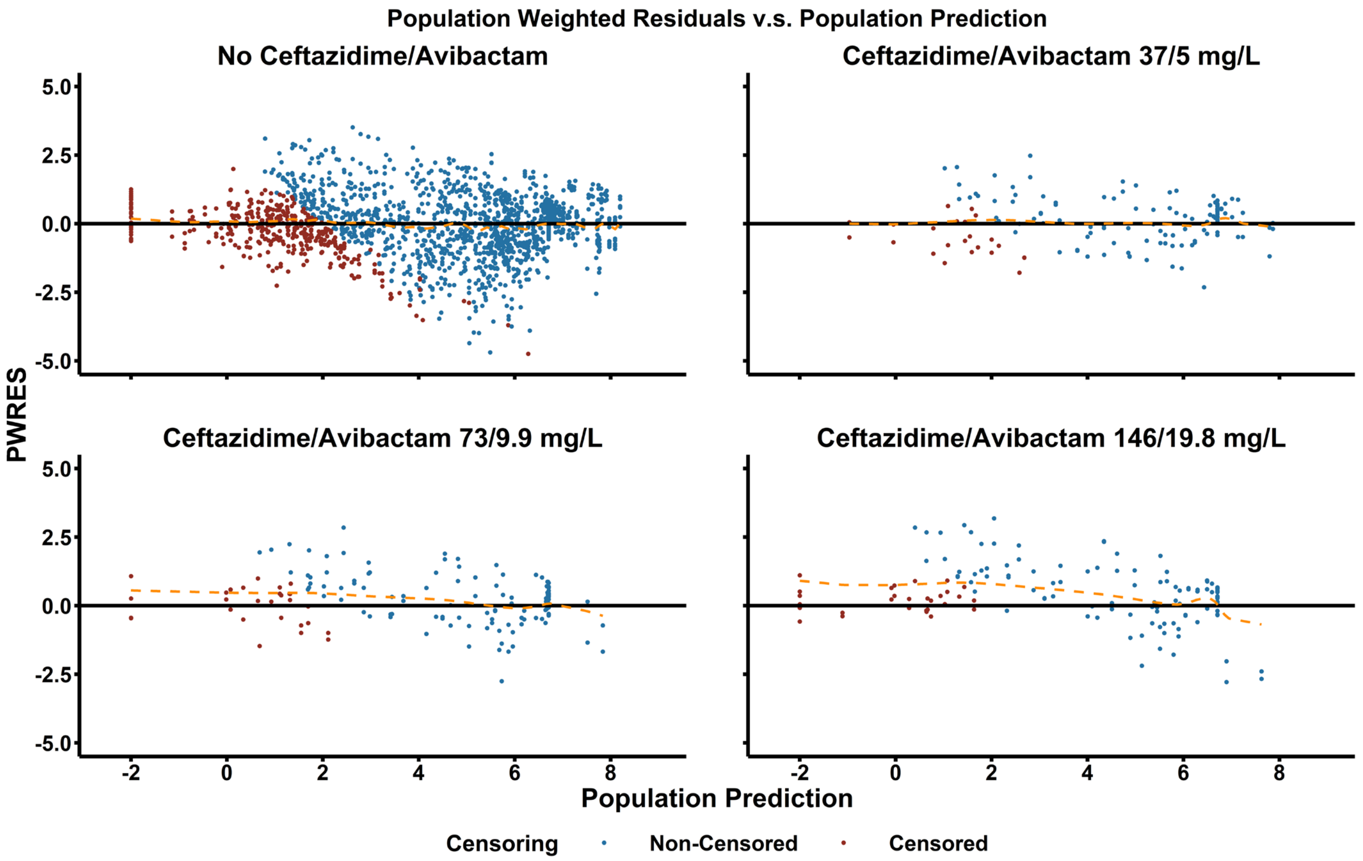

Population Weighted Residuals v.s. Population Prediction
No Ceftazidime/Avibactam
Ceftazidime/Avibactam 37/5 mg/L
Ceftazidime/Avibactam 73/9.9 mg/L
Ceftazidime/Avibactam 146/19.8 mg/L
PWRES
5.0
2.5
0.0
-2.5
-5.0
-2
0
2
4
6
8
Population Prediction
Censoring
Non-Censored
Censored

H

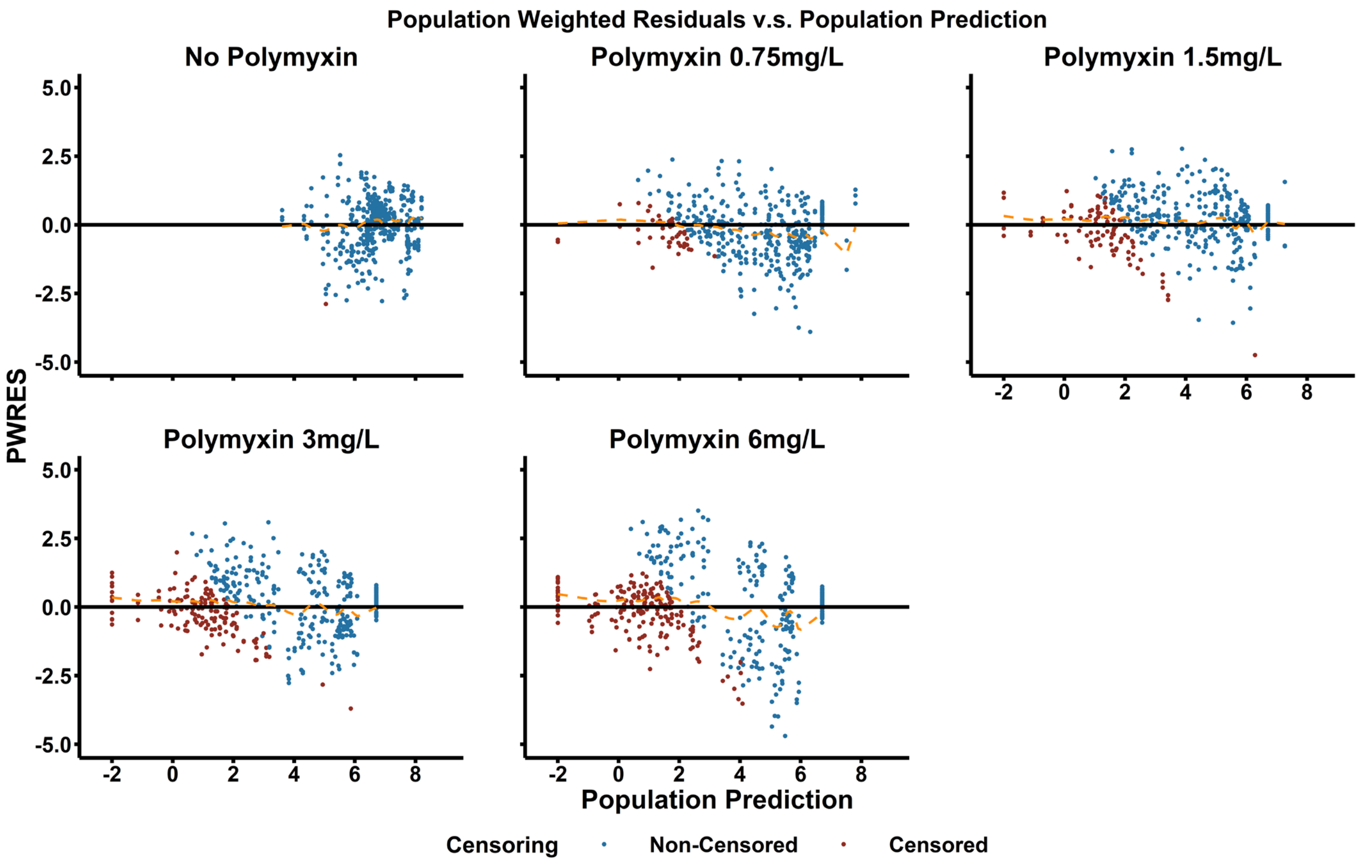

Figure S2. Population Prediction Value versus Population Weighted Residual Plots. Each plot indicates population predicted value versus population weighted residual value of total population (A), PBP7/8 knockout covariate (B), and for each monotherapy (C-H). Solid line represents the line of identity and dashed line represents the spline line indicating the general trend of data.

A

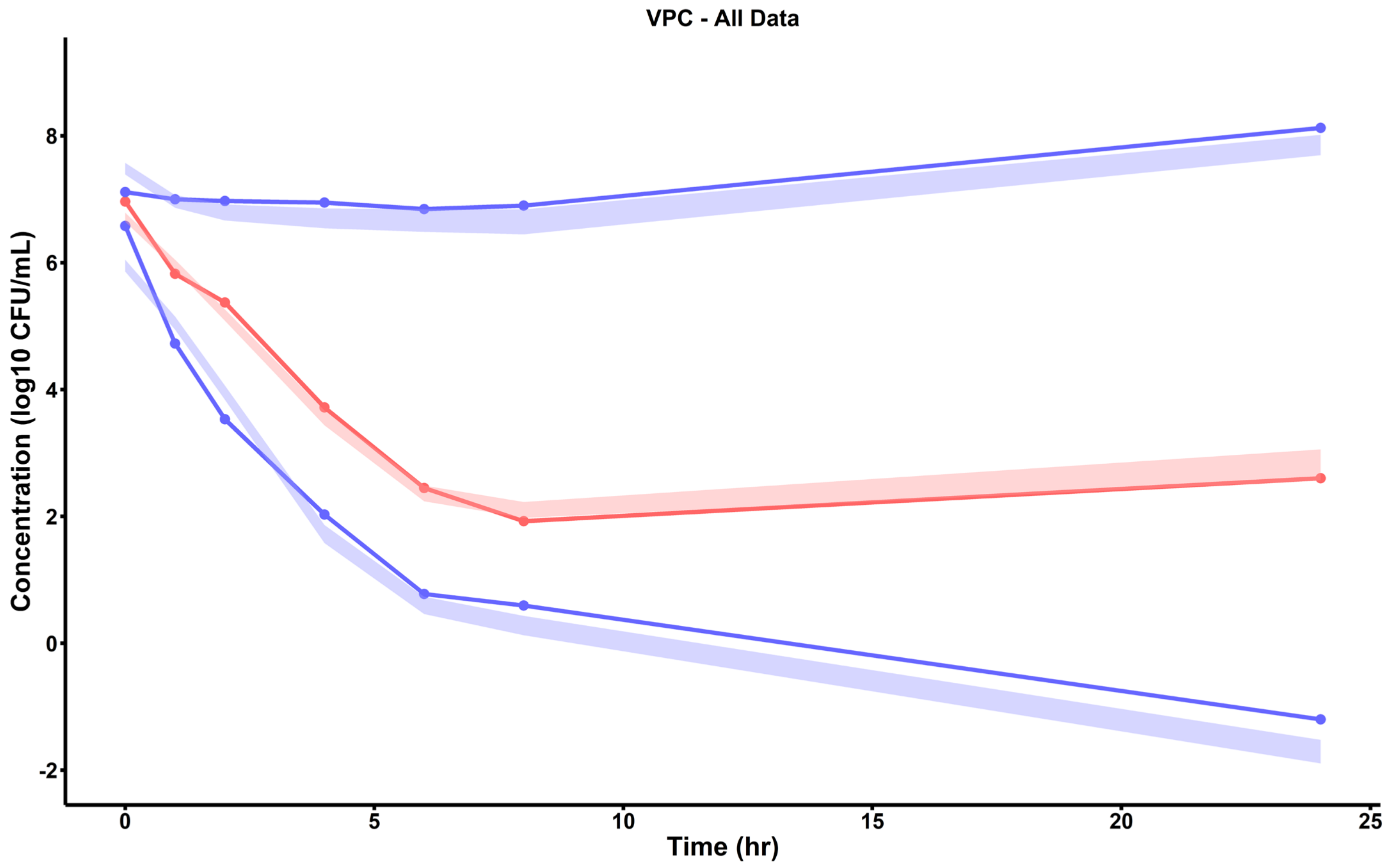

VPC - All Data
Concentration (log10 CFU/mL)
Time (hr)
-2
0
2
4
6
8
0
5
10
15
20
25

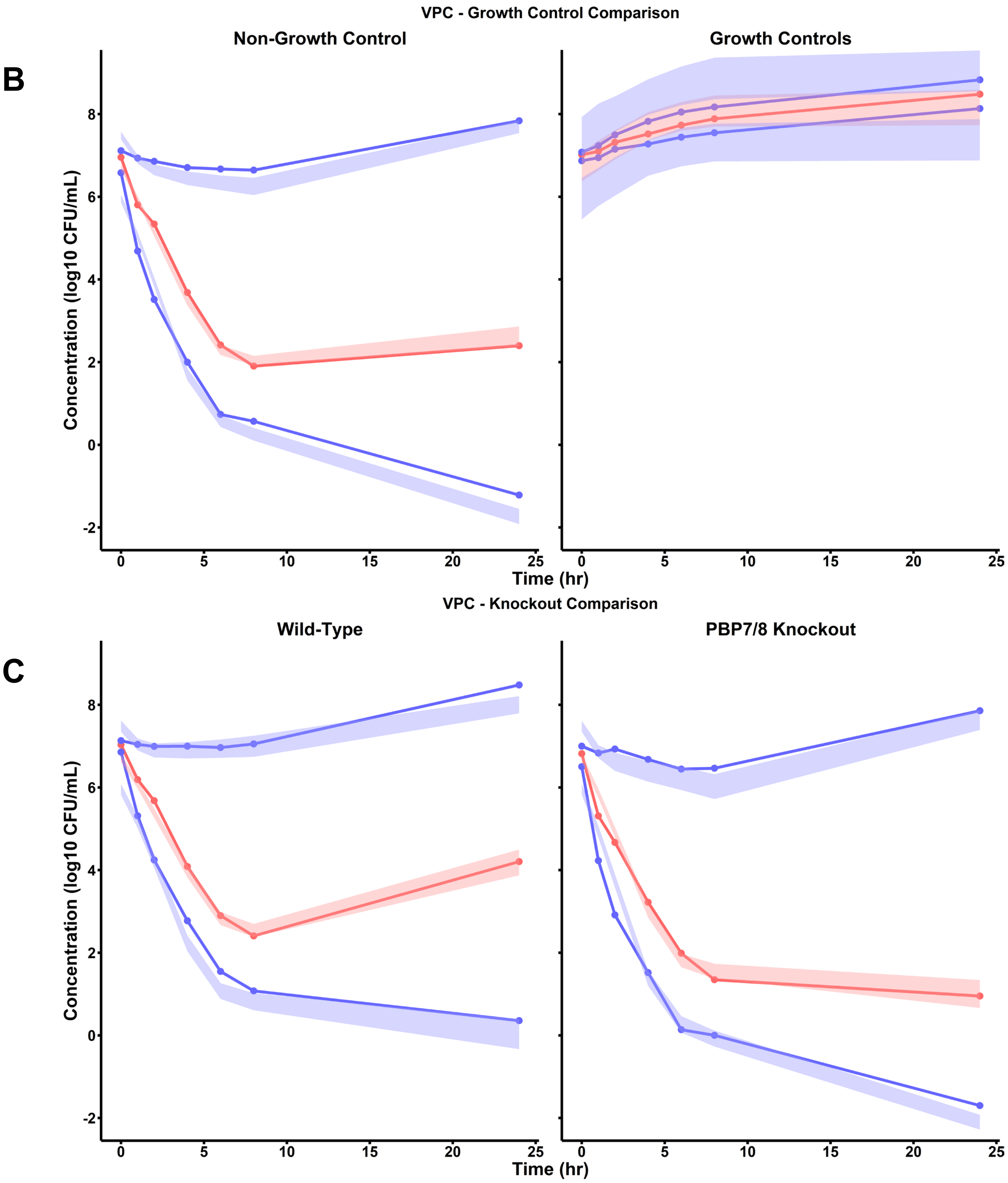

VPC - Growth Control Comparison
Non-Growth Control
Growth Controls
B
Concentration (log10 CFU/mL)
Time (hr)
VPC - Knockout Comparison
Wild-Type
PBP7/8 Knockout
C
Concentration (log10 CFU/mL)
Time (hr)

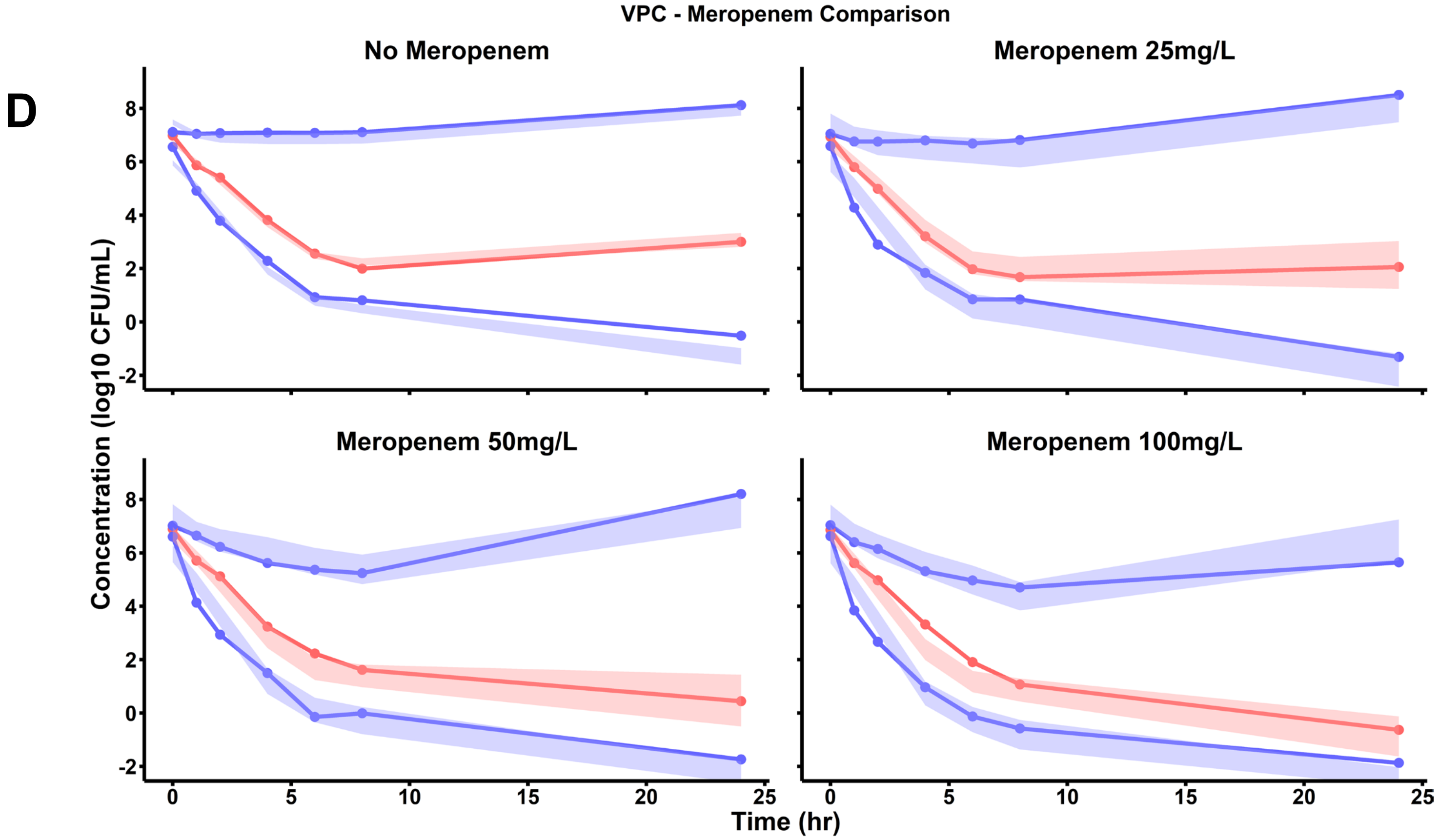

D
VPC - Meropenem Comparison
No Meropenem
Meropenem 25mg/L
Meropenem 50mg/L
Meropenem 100mg/L
Concentration (log10 CFU/mL)
Time (hr)
8
6
4
2
0
-2
0
5
10
15
20
25

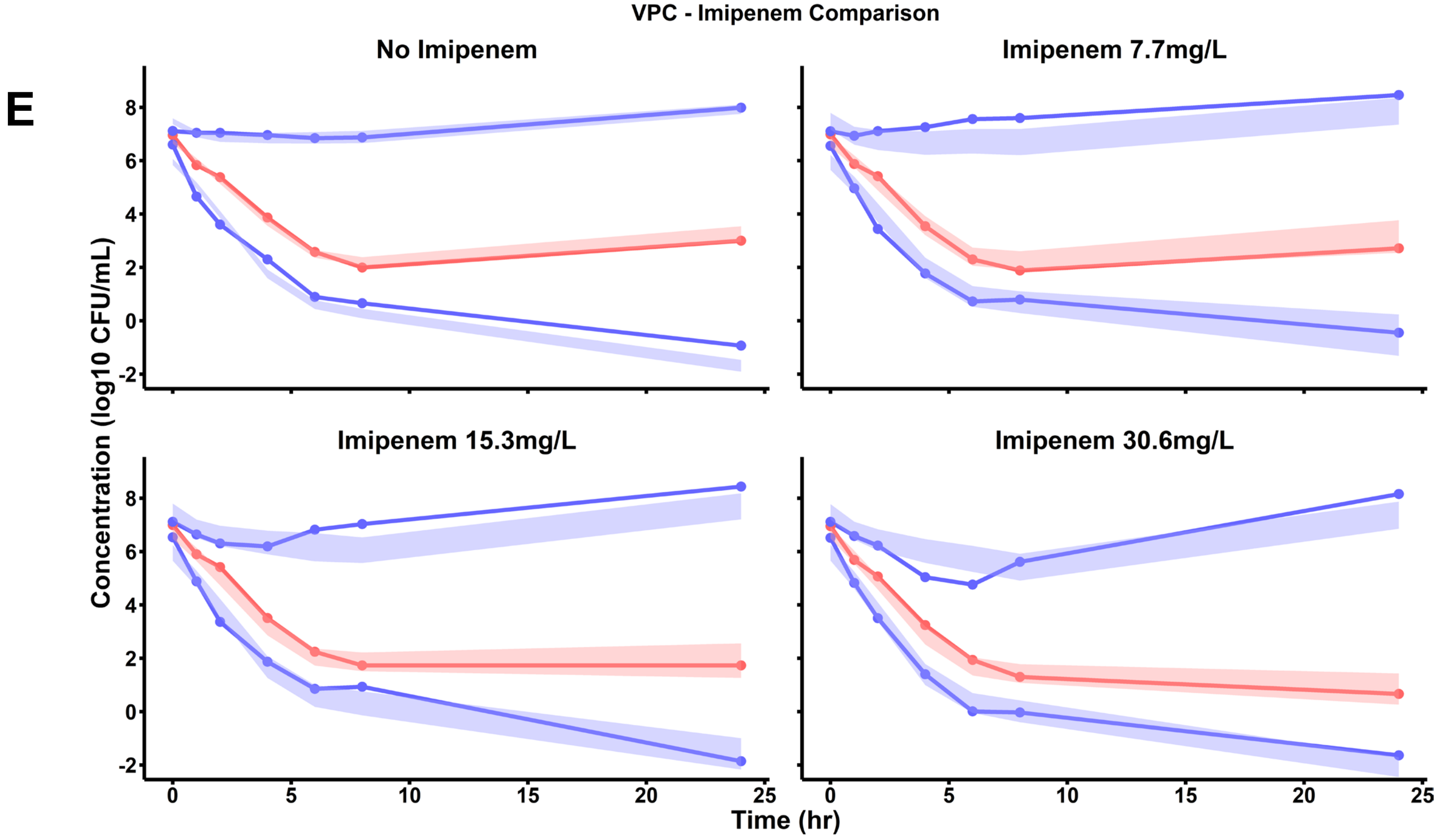

E
VPC - Imipenem Comparison
No Imipenem
Imipenem 7.7mg/L
Imipenem 15.3mg/L
Imipenem 30.6mg/L
Concentration (log10 CFU/mL)
Time (hr)
8
6
4
2
0
-2
0
5
10
15
20
25

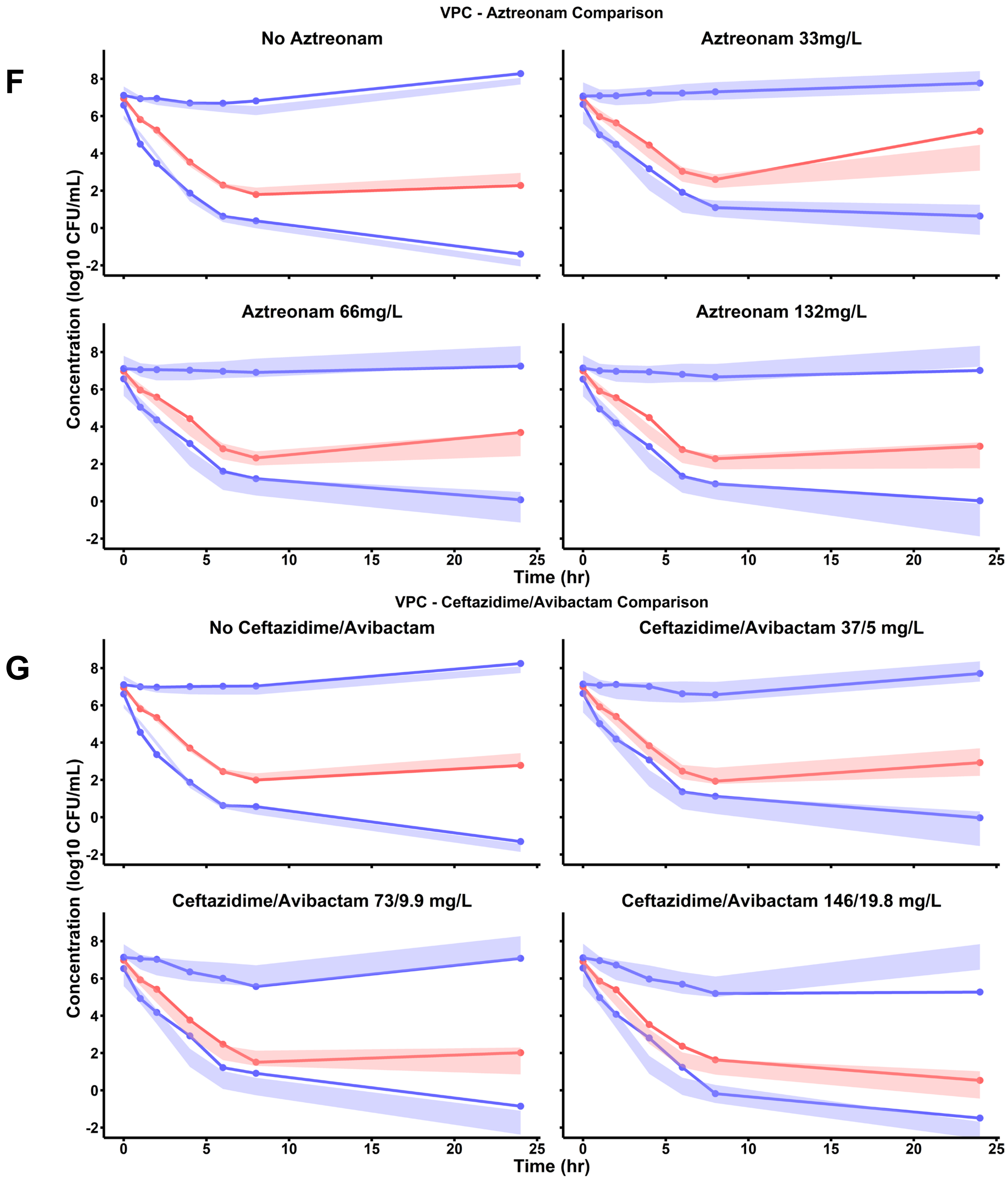
F
VPC - Aztreonam Comparison
No Aztreonam
Aztreonam 33mg/L
Aztreonam 66mg/L
Aztreonam 132mg/L
Concentration (log10 CFU/mL)
Time (hr)
G
VPC - Ceftazidime/Avibactam Comparison
No Ceftazidime/Avibactam
Ceftazidime/Avibactam 37/5 mg/L
Ceftazidime/Avibactam 73/9.9 mg/L
Ceftazidime/Avibactam 146/19.8 mg/L
Concentration (log10 CFU/mL)
Time (hr)

H

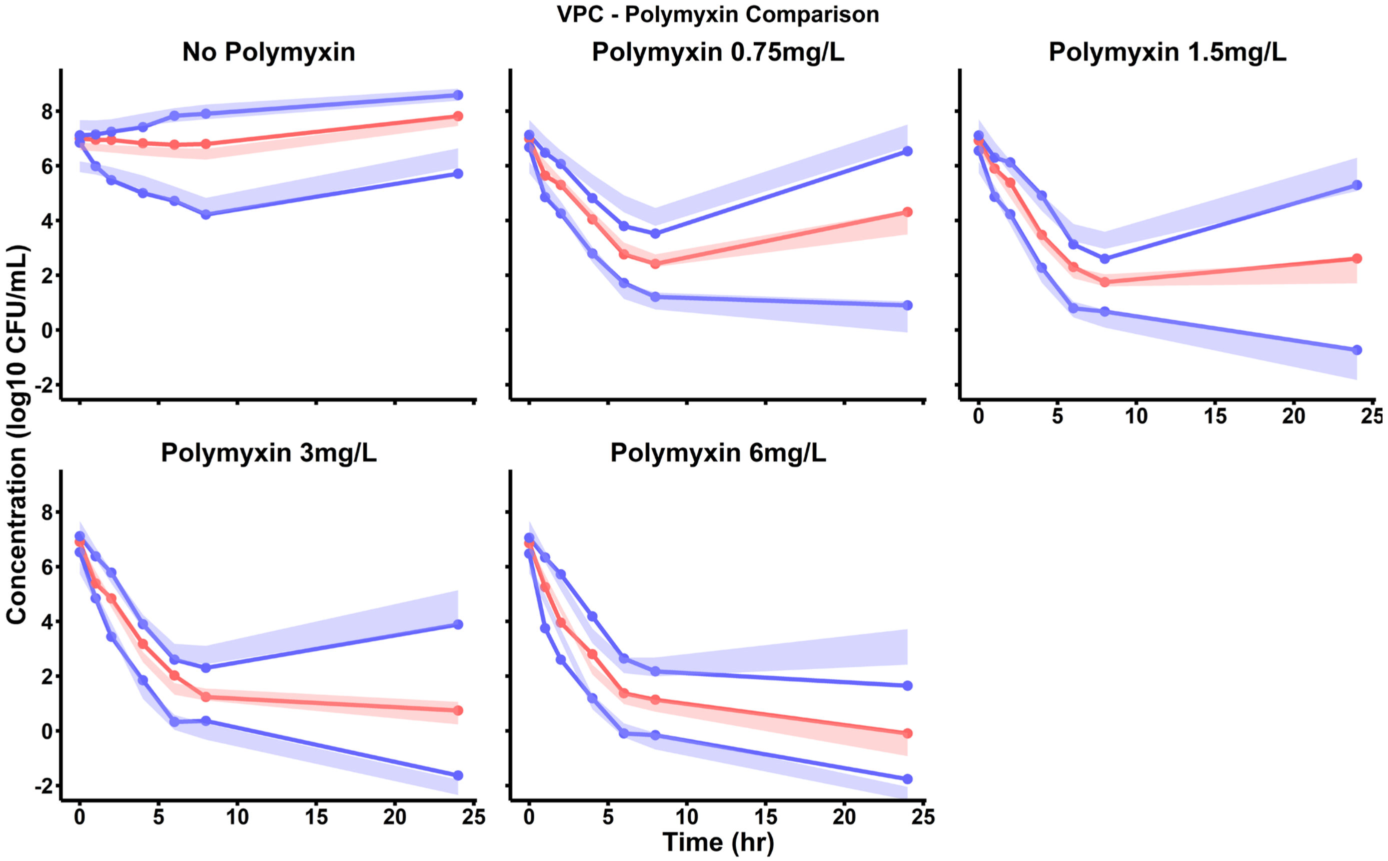

Figure S3. Visual Predictive Check of Final Pharmacodynamic Model. Each plot indicates VPC result of model for total population (A), PBP7/8 knockout covariate (B), and for each monotherapy (C-H). Dots represent the empirical percentile at the marked time. Prediction intervals for the 10th, 50th and 90th percentile is displayed as the colored areas (red for 50th percentile and blue for 10th and 90th percentile)

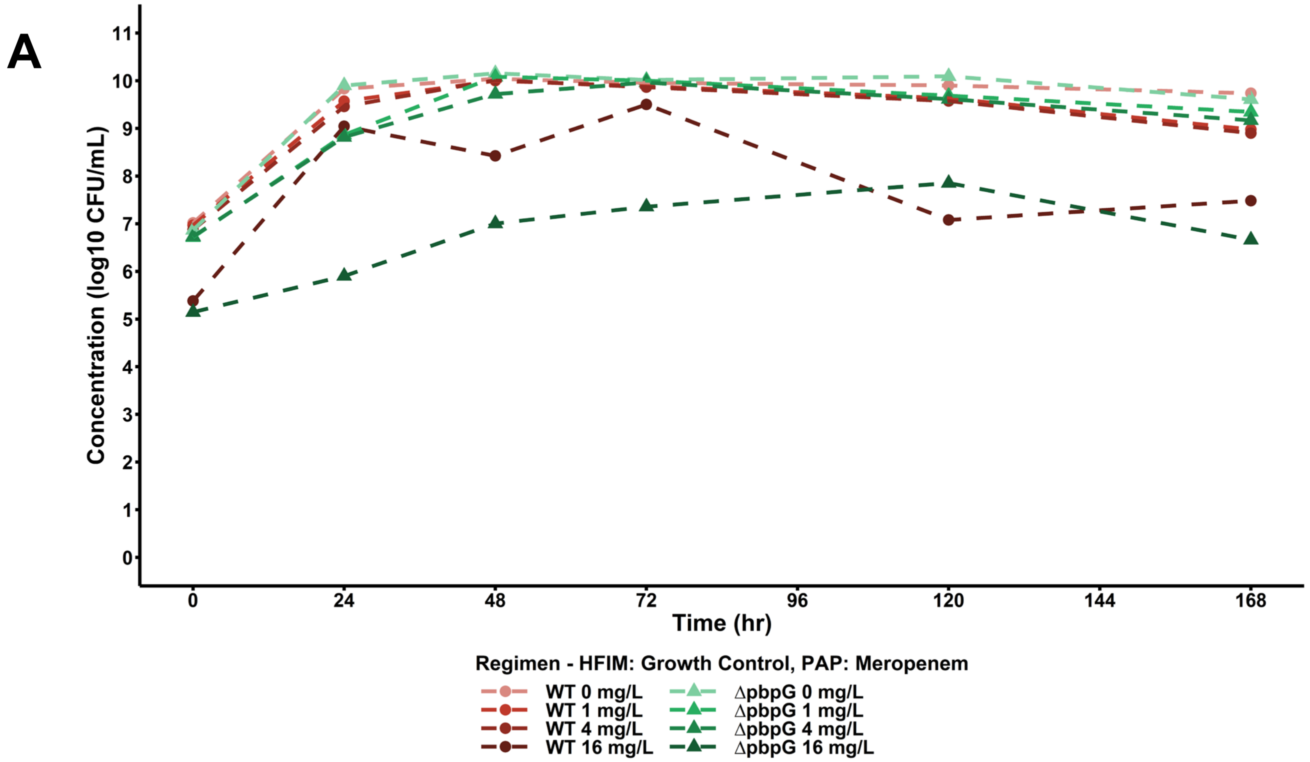
A
Concentration (log10 CFU/mL)
0
1
2
3
4
5
6
7
8
9
10
11
0
24
48
72
96
120
144
168
Time (hr)
Regimen - HFIM: Growth Control, PAP: Meropenem
WT 0 mg/L
WT 1 mg/L
WT 4 mg/L
WT 16 mg/L
ΔpbpG 0 mg/L
ΔpbpG 1 mg/L
ΔpbpG 4 mg/L
ΔpbpG 16 mg/L

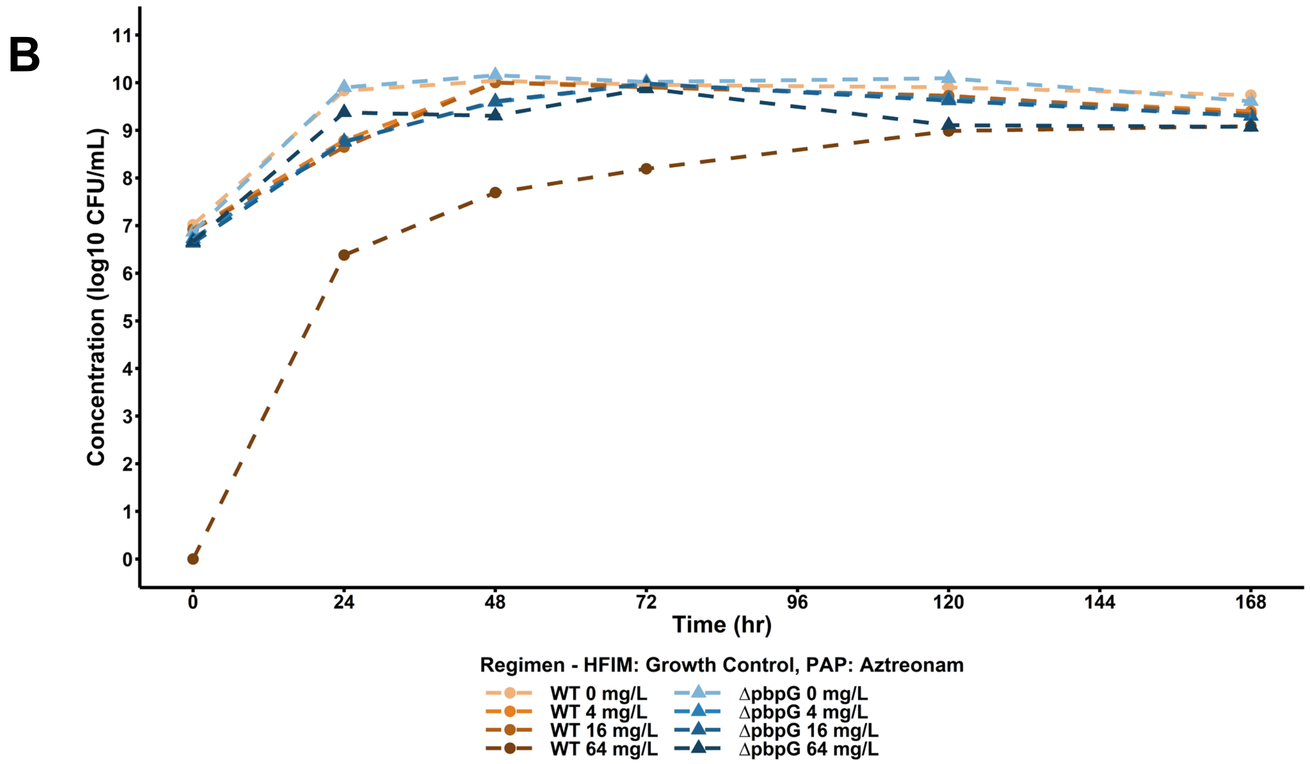
B
Concentration (log10 CFU/mL)
0
1
2
3
4
5
6
7
8
9
10
11
0
24
48
72
96
120
144
168
Time (hr)
Regimen - HFIM: Growth Control, PAP: Aztreonam
WT 0 mg/L
WT 4 mg/L
WT 16 mg/L
WT 64 mg/L
ΔpbpG 0 mg/L
ΔpbpG 4 mg/L
ΔpbpG 16 mg/L
ΔpbpG 64 mg/L

C

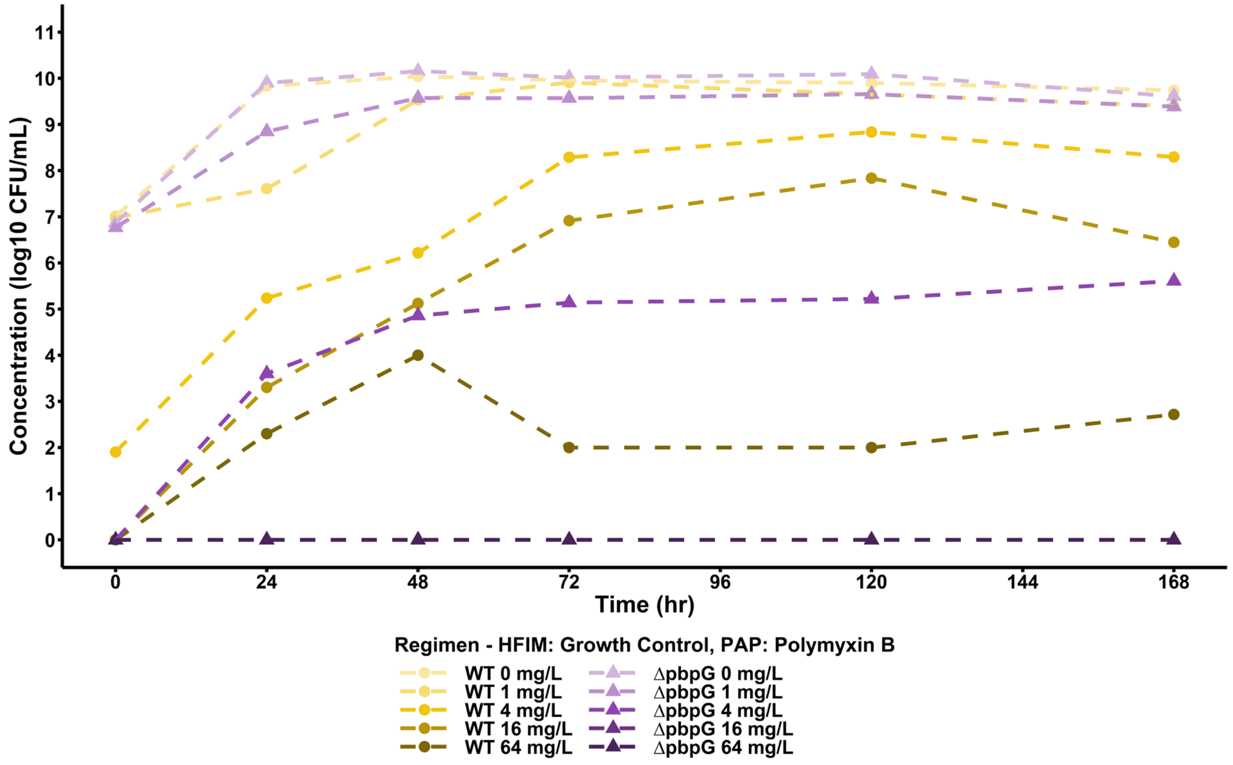

Concentration (log10 CFU/mL)
11
10
9
8
7
6
5
4
3
2
1
0
0
24
48
72
96
120
144
168
Time (hr)
Regimen - HFIM: Growth Control, PAP: Polymyxin B
WT 0 mg/L
WT 1 mg/L
WT 4 mg/L
WT 16 mg/L
WT 64 mg/L
ΔpbpG 0 mg/L
ΔpbpG 1 mg/L
ΔpbpG 4 mg/L
ΔpbpG 16 mg/L
ΔpbpG 64 mg/L

D

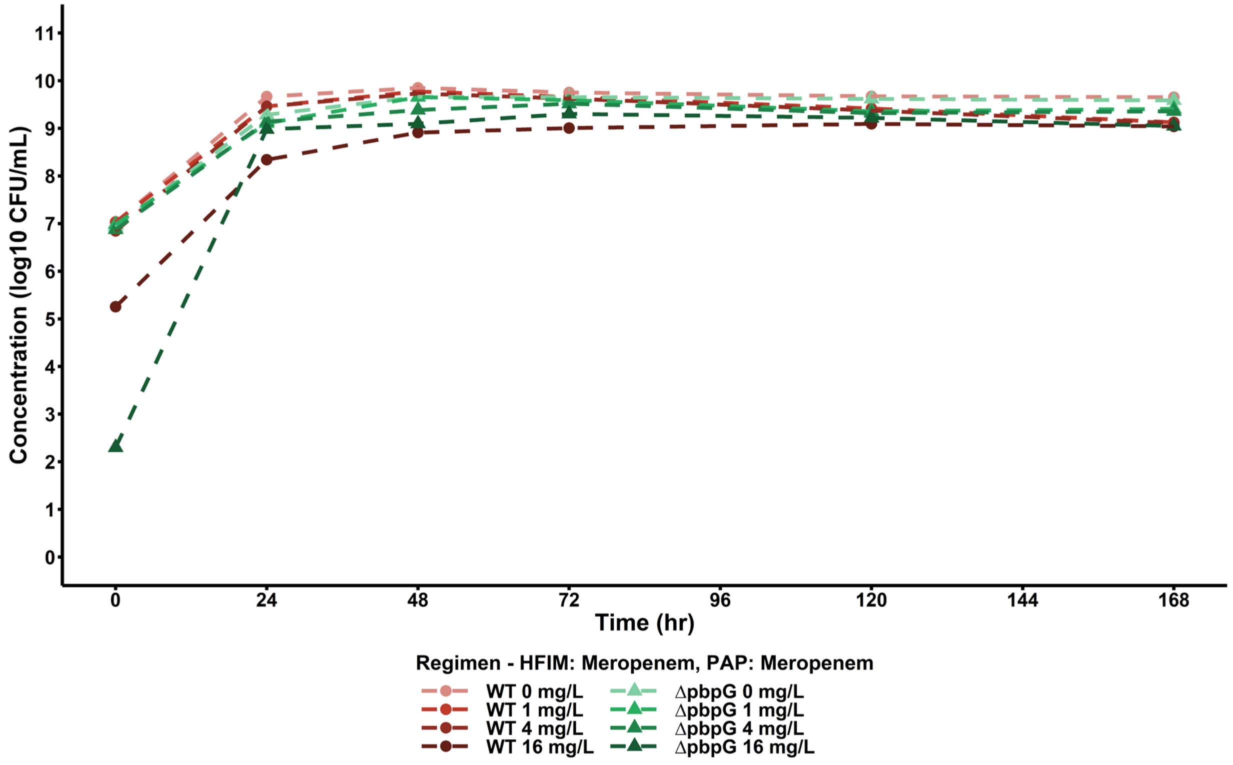

Concentration (log10 CFU/mL)
11
10
9
8
7
6
5
4
3
2
1
0
0
24
48
72
96
120
144
168
Time (hr)
Regimen - HFIM: Meropenem, PAP: Meropenem
WT 0 mg/L
WT 1 mg/L
WT 4 mg/L
WT 16 mg/L
ΔpbpG 0 mg/L
ΔpbpG 1 mg/L
ΔpbpG 4 mg/L
ΔpbpG 16 mg/L

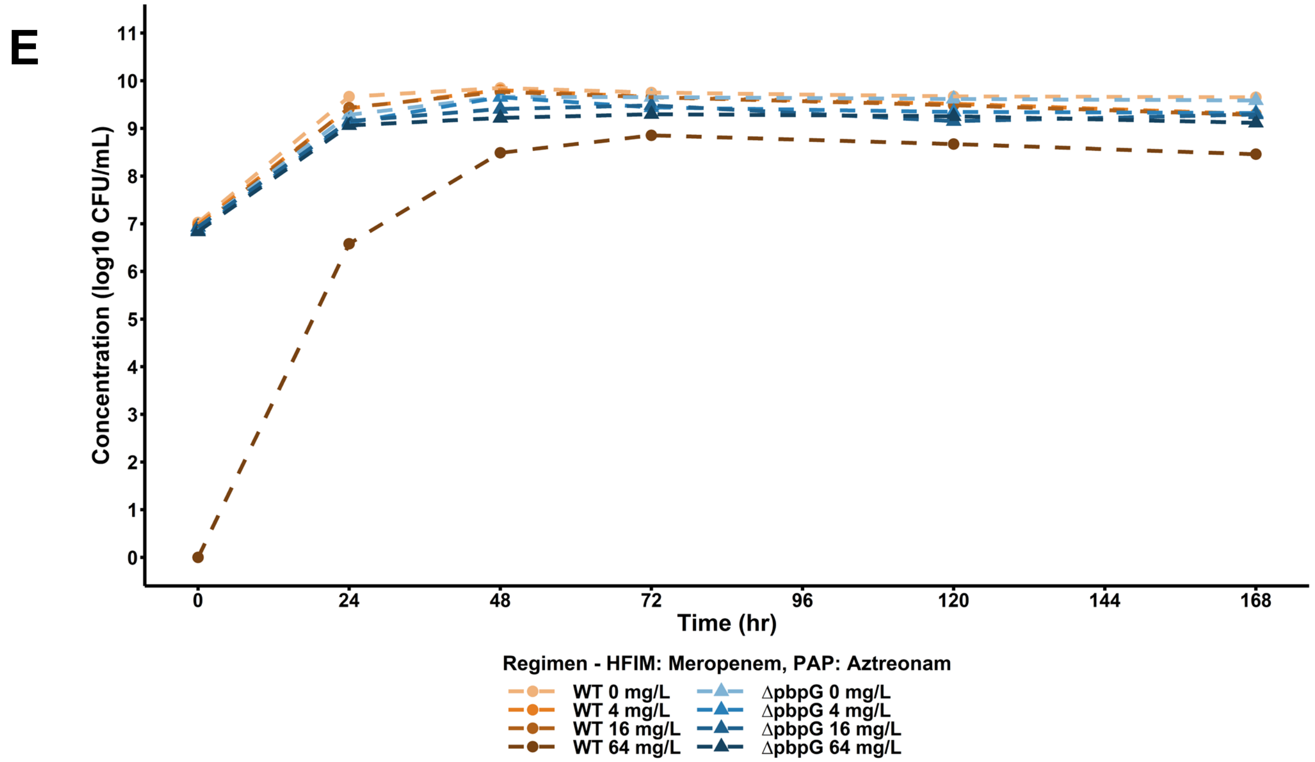
E
Concentration (log10 CFU/mL)
Time (hr)
Regimen - HFIM: Meropenem, PAP: Aztreonam
WT 0 mg/L
WT 4 mg/L
WT 16 mg/L
WT 64 mg/L
ΔpbpG 0 mg/L
ΔpbpG 4 mg/L
ΔpbpG 16 mg/L
ΔpbpG 64 mg/L

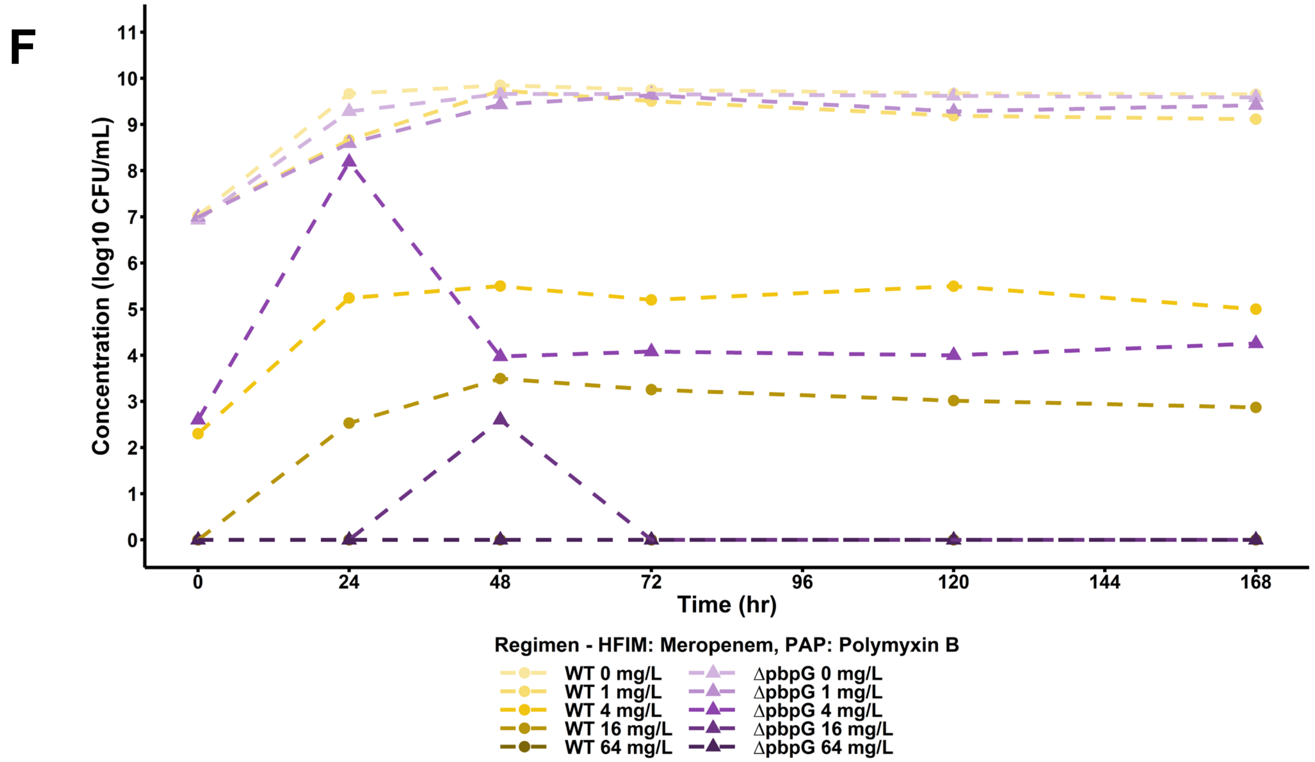
F
Concentration (log10 CFU/mL)
Time (hr)
Regimen - HFIM: Meropenem, PAP: Polymyxin B
WT 0 mg/L
WT 1 mg/L
WT 4 mg/L
WT 16 mg/L
WT 64 mg/L
ΔpbpG 0 mg/L
ΔpbpG 1 mg/L
ΔpbpG 4 mg/L
ΔpbpG 16 mg/L
ΔpbpG 64 mg/L

G

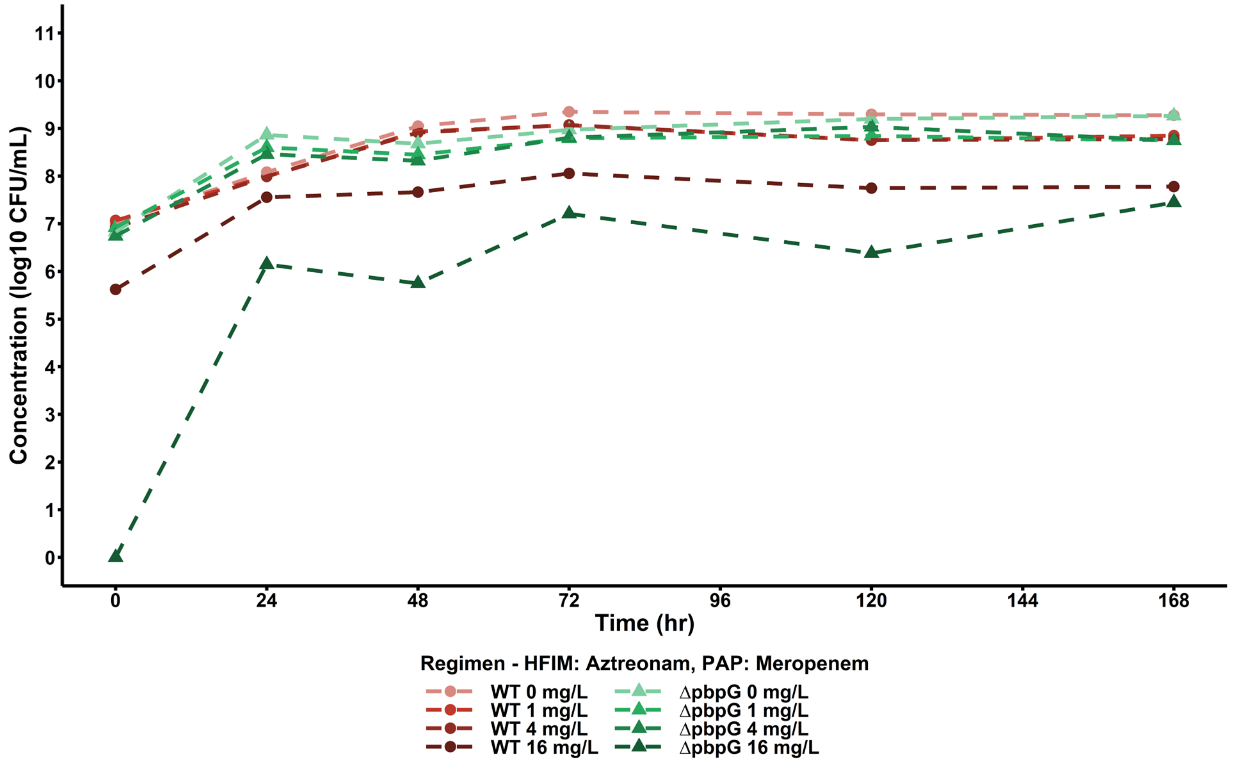
Concentration (log10 CFU/mL)
0
1
2
3
4
5
6
7
8
9
10
11
0
24
48
72
96
120
144
168
Time (hr)
Regimen - HFIM: Aztreonam, PAP: Meropenem
WT 0 mg/L
WT 1 mg/L
WT 4 mg/L
WT 16 mg/L
ΔpbpG 0 mg/L
ΔpbpG 1 mg/L
ΔpbpG 4 mg/L
ΔpbpG 16 mg/L

H

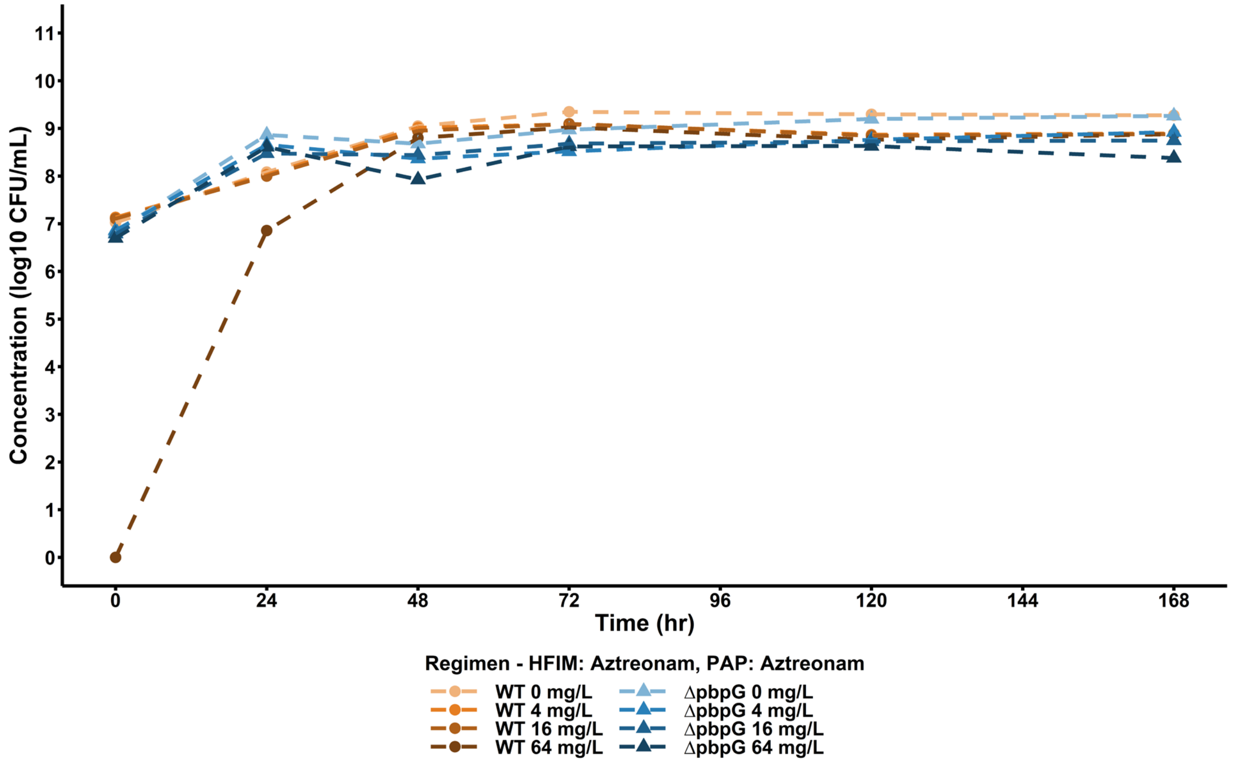
Concentration (log10 CFU/mL)
0
1
2
3
4
5
6
7
8
9
10
11
0
24
48
72
96
120
144
168
Time (hr)
Regimen - HFIM: Aztreonam, PAP: Aztreonam
WT 0 mg/L
WT 4 mg/L
WT 16 mg/L
WT 64 mg/L
ΔpbpG 0 mg/L
ΔpbpG 4 mg/L
ΔpbpG 16 mg/L
ΔpbpG 64 mg/L

I

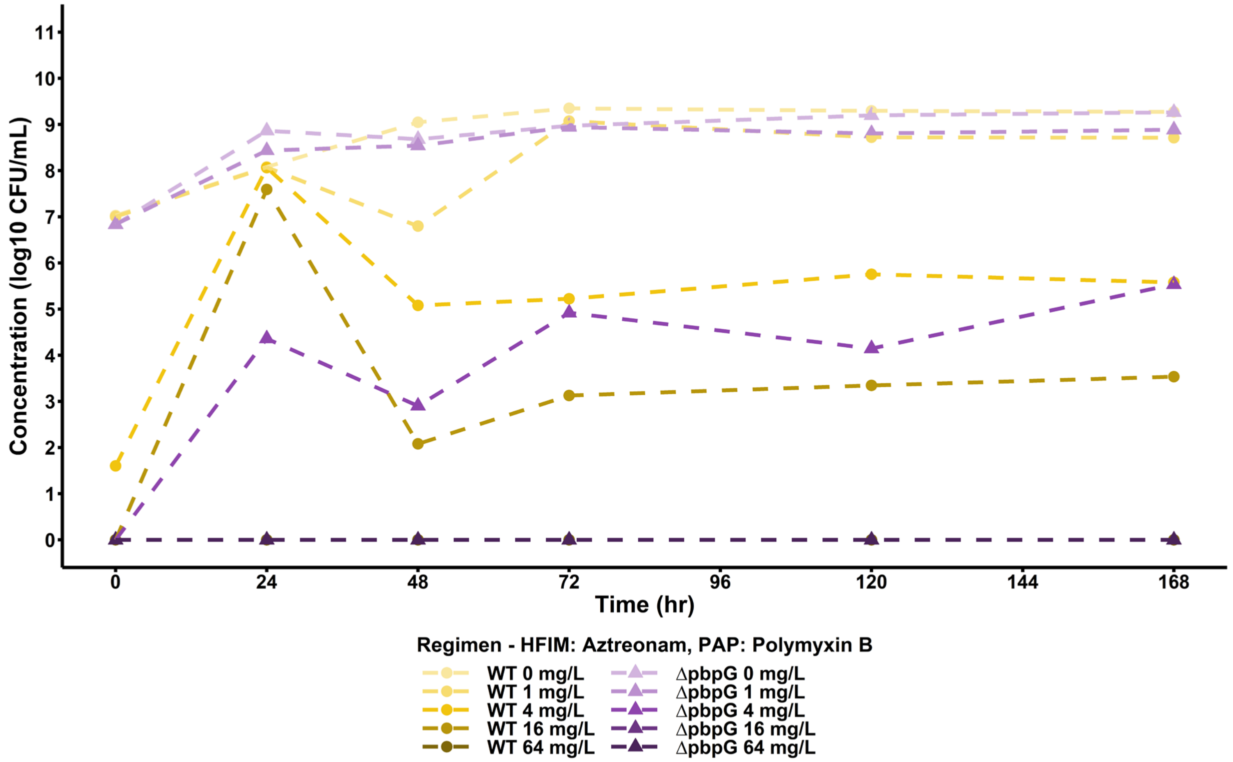
Concentration (log10 CFU/mL)
0
1
2
3
4
5
6
7
8
9
10
11
0
24
48
72
96
120
144
168
Time (hr)
Regimen - HFIM: Aztreonam, PAP: Polymyxin B
WT 0 mg/L
WT 1 mg/L
WT 4 mg/L
WT 16 mg/L
WT 64 mg/L
ΔpbpG 0 mg/L
ΔpbpG 1 mg/L
ΔpbpG 4 mg/L
ΔpbpG 16 mg/L
ΔpbpG 64 mg/L

J

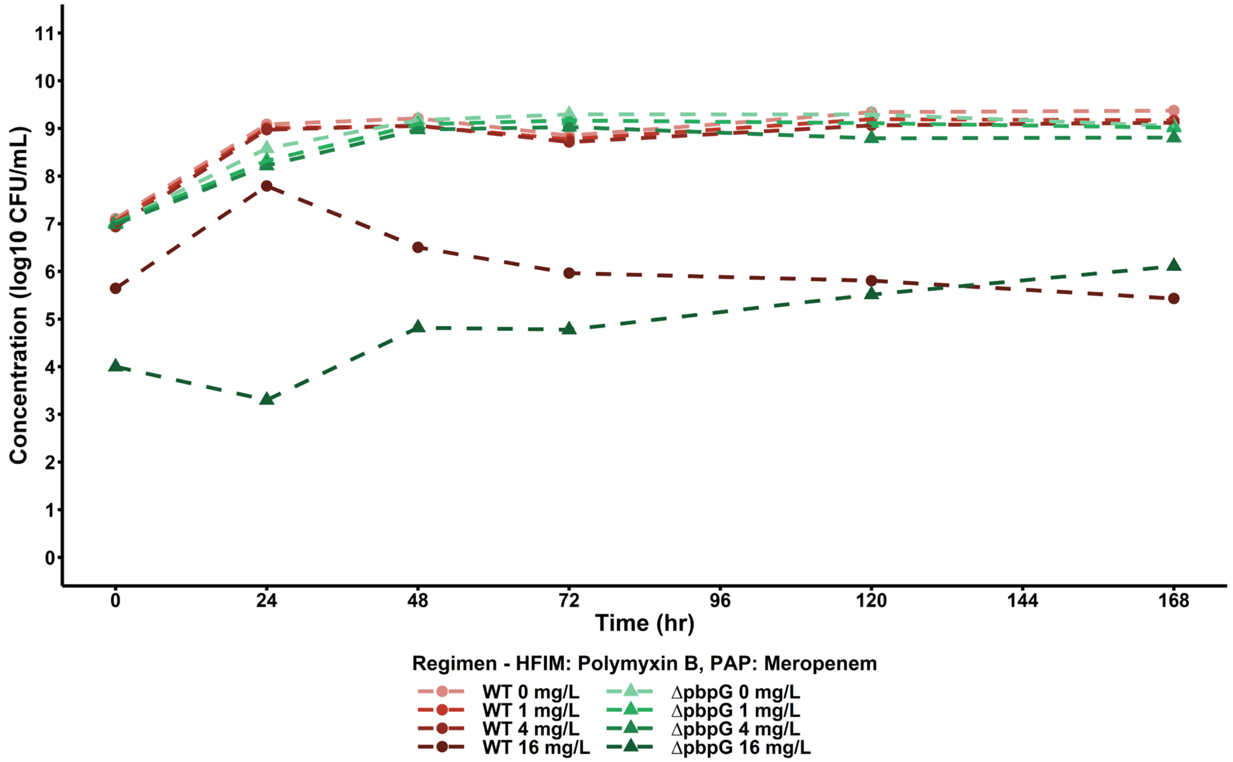
Concentration (log10 CFU/mL)
0
1
2
3
4
5
6
7
8
9
10
11
0
24
48
72
96
120
144
168
Time (hr)
Regimen - HFIM: Polymyxin B, PAP: Meropenem
WT 0 mg/L
WT 1 mg/L
WT 4 mg/L
WT 16 mg/L
ΔpbpG 0 mg/L
ΔpbpG 1 mg/L
ΔpbpG 4 mg/L
ΔpbpG 16 mg/L

K

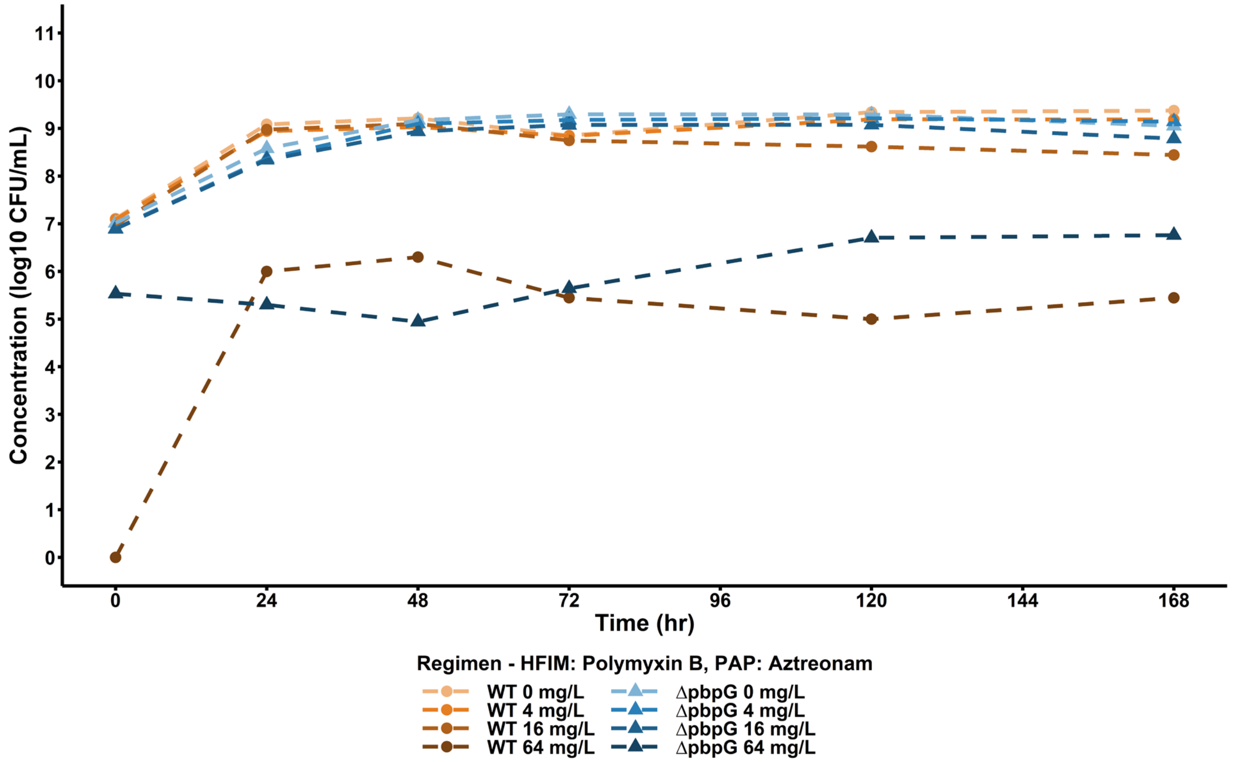

L

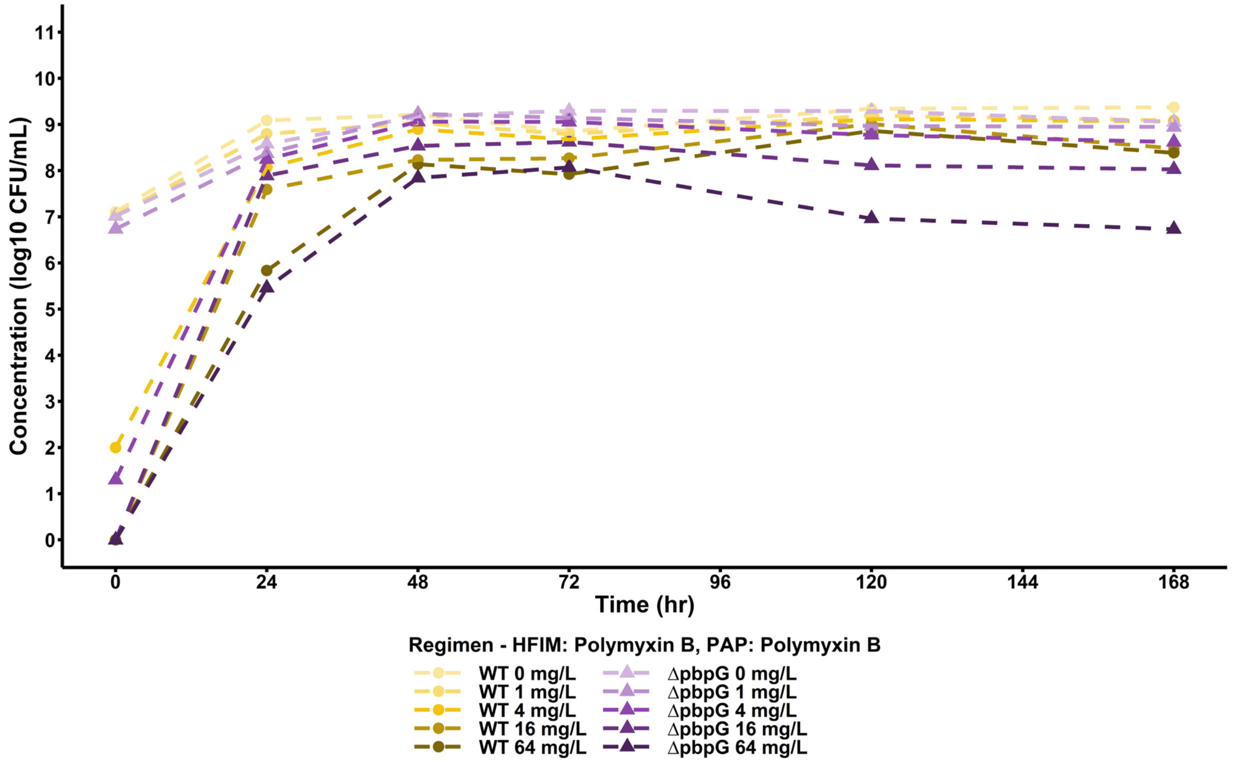

Figure S4. Hollow Fiber Infection Model Population Analysis Profile Plots. Real-time PAPs tracking the change in resistant subpopulations within the HFIM over a 168-hour period. Bacterial samples are exposed to the listed PAP antibiotic as listed in the legend. Plots are listed

as growth control (A-C), meropenem (D-F), aztreonam (G-I), and polymyxin B (J-L) with PAP exposure to meropenem, aztreonam and polymyxin B respectively.

Global Transformations

$$K_{GR,subpop} = \frac{60}{MGT_{subpop}} \quad (1)$$

$$CFU_{max} = 10^{LOGCFU_{max}} \quad (2)$$

Initial Conditions

$$INVRXN_{vol} = 1/20 \quad (3)$$

$$INOC = 10^{LOGINOC} \quad (4)$$

$$MUTR = 10^{-1*LOGMUTR} \quad (5)$$

$$CFU_{R,0} = INOC * MUTR \quad (6)$$

$$IC_S = INOC - IC_R \quad (7)$$

$$CFU_S = CFU_{S1} + CFU_{S2} \quad (8)$$

$$CFU_R = CFU_{R1} + CFU_{R2} \quad (9)$$

Bacterial Killing

$$KILL_{Drug,S} = \frac{K_{max,Drug,S} * C_{Drug}{}^{Hill_{Drug}}}{KC_{50,Drug}{}^{Hill_{Drug}} + C_{Drug}{}^{Hill_{Drug}}} \quad (10)$$

$$KILL_{Drug,R} = \frac{K_{max,Drug,R} * C_{Drug}{}^{Hill_{Drug}}}{KC_{50,Drug}{}^{Hill_{Drug}} + C_{Drug}{}^{Hill_{Drug}}} \quad (11)$$

Bacterial Subpopulation and Growth

$$CFU_{TOT} = CFU_S + CFU_R \quad (12)$$

$$RepF = 2 * \left(1 - \frac{CFU_{TOT}}{CFU_{max} + CFU_{TOT}}\right) \quad (13)$$

$$\frac{d(CFU_{S1})}{dt} = RepF * (K_{Div} * CFU_{S2}) - (K_{GR,S} * CFU_{S1}) - (KILL_{MER,S} + KILL_{IMI,S} + KILL_{ATM,S} + KILL_{CEF,S} + KILL_{PMB,S}) \quad (14)$$

$$CFU_{S1}(0) = IC_S \quad (15)$$

$$\frac{d(CFU_{S2})}{dt} = (K_{GR,S} * CFU_{S1}) - (K_{Div} * CFU_{S2}) - (KILL_{MER,S} + KILL_{IMI,S} + KILL_{ATM,S} + KILL_{CEF,S} + KILL_{PMB,S}) \quad (16)$$

$$CFU_{S2}(0) = 0 \quad (17)$$

$$\frac{d(CFU_{R1})}{dt} = RepF * (K_{Div} * CFU_{R2}) - (K_{GR,R} * CFU_{R1}) - (KILL_{MER,R} + KILL_{IMI,R} + KILL_{ATM,R} + KILL_{CEF,R} + KILL_{PMB,R}) \quad (18)$$

$$CFU_{R1}(0) = IC_R \quad (19)$$

$$\frac{d(CFU_{R2})}{dt} = (K_{GR,R} * CFU_{R1}) - (K_{Div} * CFU_{R2}) - (KILL_{MER,R} + KILL_{IMI,R} + KILL_{ATM,R} + KILL_{CEF,R} + KILL_{PMB,R}) \quad (20)$$

$$CFU_{R2}(0) = 0 \quad (21)$$

Figure S5. Model Equations
